# Distinct genetic origins of eumelanin levels and barring patterns in cichlid fishes

**DOI:** 10.1371/journal.pone.0306614

**Published:** 2024-07-08

**Authors:** A. Allyson Brandon, Cassia Michael, Aldo Carmona Baez, Emily C. Moore, Patrick J. Ciccotto, Natalie B. Roberts, Reade B. Roberts, Kara E. Powder

**Affiliations:** 1 Department of Biological Sciences, Clemson University, Clemson, South Carolina, United States of America; 2 Department of Biological Sciences, Genetics and Genomics Academy, North Carolina State University, Raleigh, North Carolina, United States of America; 3 Department of Biology, Warren Wilson College, Swannanoa, North Carolina, United States of America; University of Iceland, ICELAND

## Abstract

Pigment patterns are incredibly diverse across vertebrates and are shaped by multiple selective pressures from predator avoidance to mate choice. A common pattern across fishes, but for which we know little about the underlying mechanisms, is repeated melanic vertical bars. To understand the genetic factors that modify the level or pattern of vertical barring, we generated a genetic cross of 322 F_2_ hybrids between two cichlid species with distinct barring patterns, *Aulonocara koningsi* and *Metriaclima mbenjii*. We identify 48 significant quantitative trait loci that underlie a series of seven phenotypes related to the relative pigmentation intensity, and four traits related to patterning of the vertical bars. We find that genomic regions that generate variation in the level of eumelanin produced are largely independent of those that control the spacing of vertical bars. Candidate genes within these intervals include novel genes and those newly-associated with vertical bars, which could affect melanophore survival, fate decisions, pigment biosynthesis, and pigment distribution. Together, this work provides insights into the regulation of pigment diversity, with direct implications for an animal’s fitness and the speciation process.

## Introduction

Coloration of animals has long fascinated both scientists and non-scientists alike. For centuries, scientists have asked questions about the underlying genetic control, diversity of variation, and ecological relevance of changes in pigmentation [1–3]. The rich collection of hues, spots, stripes, and bars of animals integrate both natural and sexual selective pressures [4–7]. Pigmentation patterns are related to crypsis and predator avoidance, mate choice, color-mediated aggression, social dominance and competitive interactions, nutritional health, and collective animal behaviors such as schooling or shoaling in fishes [4, 6–13]. Through this, these traits directly affect reproductive success, fitness, and speciation [14], and the ultimate result is an incredible array of color pattern variation across animals.

One clade with notable variation in pigmentation is cichlid fishes, which have undergone a rapid and extensive adaptive radiation [15, 16]. Cichlids exhibit dramatic variation in their coloration, with variation due to species, sex, and geography [6, 17]. The evolution of pigmentation is particularly important in cichlids, where sexual selection on divergent nuptial coloration appears to maintain pre-mating reproductive isolation among the most recently evolved species [18]. Much work has been done to begin to understand the molecular origins of the rich palette found across cichlids. Various genomic approaches have identified genetic loci that regulate black blotches [19, 20], dark horizontal stripes [21], yellow egg spots [22, 23], black and yellow coloration of the fins [24, 25], golden morphs [26], albinism [27], and even modularity in patterns across the flank [28]. However, one pigment phenotype that is understudied is the most common pigment pattern, dark vertical barring [16]. In contrast to horizontal stripes, whose presence and absence is controlled by a master switch gene *agouti-related peptide 2* (*agrp2*, also known as *asip2b*) [21], the presence of barring in cichlids is predicted to be polygenic [29]. These darkly pigmented bars are primarily due to a population of melanin producing cells called melanophores. Melanophores originate from trunk neural crest cells, as do other pigment cells in teleosts including xanthophores that generate red/yellow pigment and iridophores which are reflective [8].

**Fig 1:**
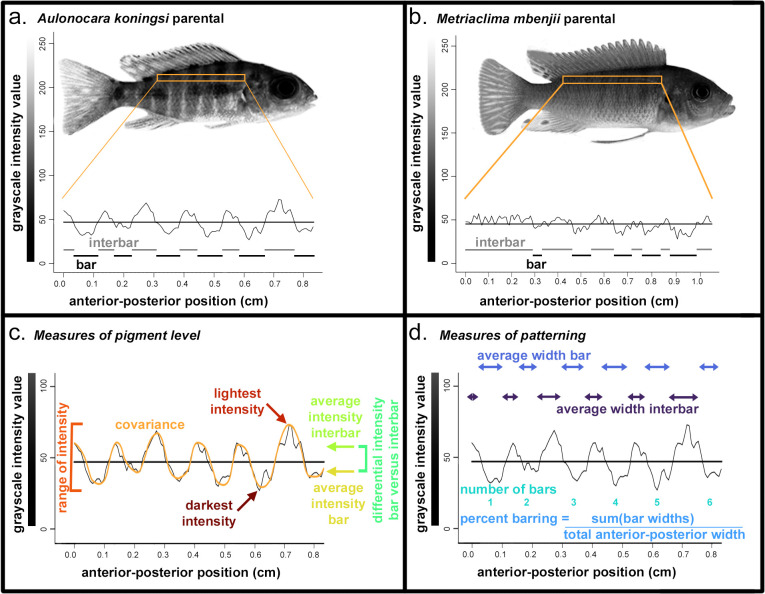
Parental species and measures of variation in barring pattern and pigment level. Representative parental for (a) *Aulonocara koningsi* and (b) *Metriaclima mbenjii*, including quantification of the region in the orange rectangle into grayscale values. The horizontal bar in graphs in (a) and (b) are the average grayscale intensity value, which was calculated for each individual and used to characterize bar and interbars, indicated by black and gray marks, respectively, in (a) and (b). From grayscale plots for each individual, measures of (c) eumelanin pigment level and (d) bar patterning were calculated as visualized, using the individual shown in Fig 1A as an example. Colors in (c) and (d) match colors used in Fig 3.

Here, we sought to determine the underlying genetic regulators of variation in vertical bar pigmentation. To accomplish this, we generated a genetic mapping cross of two Lake Malawi cichlids with alternate barring phenotypes. Animals of these two species are similar in size, and their body pigmentation is largely based on differences in eumelanin levels from melanophores without drastic differences in other pigmentation (S1 Fig for color figures and Fig 1A and 1B for black and white figures). The two species inhabit different “rock” and “sand” habitats [17], are consistently resolved into distinct clades in whole-genome phylogenetic analyses [30], and are regularly co-housed in the lab without interbreeding. Thus, they are not thought to naturally hybridize. *Aulonocara koningsi* has high-contrast bars across its body, and *Metriaclima mbenjii* has fewer and fainter bars, with little contrast between bars and the background pigment levels (Fig 1A and 1B). It is currently unclear if the alternate barring patterns in these species are primarily driven by sexual or natural selection. Vertical bars are more common in shore-dwelling cichlid species where they can provide camouflage by disrupting the outline of the animal [6, 31, 32]; this is consistent with *Aulonocara* living within an open, sandy region while *Metriaclima* is generally found is a more complex rocky region [17]. However, pigmentation patterns are proposed as largely driven by sexual selection, intrasexual competition, and species recognition in cichlids [18, 33, 34], and the amount of vertical barring has been shown to directly impact female choice of a mate in both cichlid [35] and swordtail fishes [36].

Regardless of the cause of variation in barring, these species enable us to identify the genetic sources of variation in this trait. By crossing two species that both display vertical barring, we set out to identify factors that alter the intensity and spacing of these bars, rather than master regulators governing their presence. In particular, we expected that one set of genomic regions would regulate where melanophores were located and the pattern of the bars, while a separate set of genes would independently regulate the levels of black/brown eumelanin being produced and dispersed from melanophores. We identify genetic intervals with candidate genes that are redeployed across vertebrates to regulate barring in addition to other pigment phenotypes, as well as a series of additional genetic regions that are novel regulators of barring. Together, these data provide insights into the genetic and molecular underpinnings of pigment biodiversity, which lies at the intersection of a series of selective pressures that shape an animal’s ecology and evolution.

## Materials and methods

### Experimental cross

All animal care was conducted under protocol 14-101-O, as approved by the IACUC at North Carolina State University. A single F_1_ hybrid family of seven individuals was generated from a cross between a single female *Metriaclima mbenjii* and two male *Aulonocara koningsi*. The inadvertent inclusion of two grandsires resulted from an unexpected fertilization, as these animals externally fertilize. Two F_1_ males and two F_1_ females were intercrossed to produce the F_2_ generation. Of these, both males and one female shared a sire, while the remaining female had the other sire. We discuss how this was accounted for during genotyping in the section *Genotyping and linkage map generation*. F_2_ hybrids were raised in density-controlled aquaria and with standardized measured feedings until around sexual maturity (five months of age), at which time they were sacrificed for analysis and weighed. For each individual, the standard length of the animal was measured from a photograph using FIJI software (version 2.9.0) [37], as the number of pixels between the snout and the caudal peduncle; the number of pixels was then converted into absolute length in centimeters using a scale bar included in each picture. *Aulonocara* parentals were slightly smaller than *Metriaclima* parentals, but neither parental population was statistically distinct in mass or length from the population of their F_2_ hybrids (S1 Table). The sex of each animal was determined based on a combination of gonad dissections at sacrifice and genotype at an XY locus on LG7 that solely determined sex in this cross [38, 39]. Sex was omitted for animals with ambiguity (e.g. under-developed gonads) or discrepancies between these calls (8.77% of animals), resulting in a set of 479 hybrids with 48.74% females.

### Imaging and filtering of data set

Images were taken of animals that were freshly sacrificed via cold buffered 100 mg/L MS-222. Euthanasia in cold solutions relaxed chromatophores in the skin to maximize black/brown eumelanin-based pigmentation [28]. While this approach enables analysis of the contribution of melanophores to barring, it precludes examination of other pigment cell types such as xanthophores or iridophores. Whole fish photographs were taken using a uniform setup, with standard lighting conditions in a lightbox with a mirrorless digital camera (Olympus). All images included a scale bar and a gray scale color standard. Images were color balanced in Adobe Photoshop (version 22.0.0 or after) using the black and white segments of the color standard. From the total data set of 10 parentals of *Aulonocara koningsi*, 10 parentals of *Metriaclima mbenjii*, and 479 F_2_ hybrids, we omitted fish that exhibited two pigmentation phenotypes. First, *Metriaclima mbenjii* has a high percentage of animals that carry the ‘orange blotch’ (OB) phenotype, which results in marbled melanophore blotches rather than distinct barring [17, 19, 20]. To enable analysis of barring patterns, we therefore removed 4 OB *Metriaclima mbenjii* parentals and 96 OB F_2_ hybrids. We further removed 65 hybrids that were heavily melanic to the degree that the eye was not distinguishable from the head or flank and thus anatomical landmarks used for additional processing were not visible. The final data set after this filtering included 10 *Aulonocara koningsi* parentals, 6 *Metriaclima mbenjii* parentals, and 322 F_2_ hybrids (48.97% female).

### Isolation and quantification of pigmented region

Images were rotated so a horizontal guideline aligned the midline of the caudal peduncle and the tip of the snout. A second horizontal guideline was added at the top of the caudal peduncle. From this image, a region was extracted for the remainder of the analysis (indicated by orange outlines in Fig 1A and 1B). This region was 10 pixels high with the ventral side aligned with the guide at the top of the caudal peduncle, the opercle at the anterior end, and the dorsal fin on the posterior end (Fig 1A and 1B). This standardized region of the body was chosen as it has a barring pattern representative of the entire flank and avoids areas that included the pectoral fin in a portion of images, which introduced variation in measurements of pigmentation.

The image of the isolated region was uploaded to FIJI software (version 2.9.0) [37], where it was converted to 32-bit grayscale. Following [40, 41], the Plot Profile command in FIJI was used to convert the image to a numerical gray value from 0 (pure black) to 255 (pure white), averaging the values of the 10 pixels in each column (Fig 1). Isolated regions had an average width of 174 ± 36 pixels, which equated to 1.26 ± 0.26 centimeters or 30.6 ± 3.8% of the total length (snout to caudal peduncle) of the animal.

### Quantification of melanic traits

Outputted data from Plot Profile in FIJI were analyzed in either R or with a custom perl script available at https://github.com/kpowder/Biology2022. The perl script used two criteria to define bars and interbars (defined as the region between bars) (Fig 1A and 1B) based on empirical testing of four *Aulonocara koningsi* parentals, four *Metriaclima mbenjii* parentals, and four F_2_ hybrids, all randomly-chosen. Both cutoffs described below were selected as they accurately represented the barring pattern that was observed by eye on this test data set (S2 Fig). First, we used the average intensity value to define bar regions, with gray intensity value less than (i.e., darker than) the average considered within a bar, and gray intensity value greater than this average considered within an interbar (Fig 1A and 1B and S2 Fig). Second, to minimize overcounting of bars due to variation in pigment intensity from one pixel to the next, we required a bar to have at least 5 sequential pixels with intensity values below the average, and define the end of the bar as 5 pixels in a row above the average gray intensity value.

From the Plot Profile data and output of the perl script, we calculated seven measures related to variation in the levels of eumelanin produced: darkest intensity, lightest intensity, range of intensity, covariance of intensity measure with anterior-posterior position, the average intensity of bars, the average intensity of interbars, and the differential intensity between bars and interbars (Fig 1C). We note that an increased range of intensity, covariance, and differential intensity between bars and interbars are characteristic of animals with more discrepancy between bars and interbars (that is, highly melanic bars on very pale backgrounds). We also calculated four measures based around variation in the pattern of what regions of the body had bars: the total number of bars, the average width of bars, the average width of interbars, and the percent of barring, measured as the total length of regions classified as bars divided by the total length of the isolated region (Fig 1D).

### Statistical analysis of pigment measures

To remove the effects of size on pigment phenotypic measures, all measurements were normalized to the standard length using a dataset including both parentals and hybrids. To do this, we first generated a linear model of each measure compared to standard length, then calculated the residual for each individual compared to the regression line. These residual data represent an allometry-free measure that account for factors such as genetics that serve as a source of phenotypic variability. Analyses including linear normalization, ANOVAs, Tukey’s Honest Significant Difference post-hoc tests, and Pearson’s correlations were conducted in R (version 3.5.2 or higher).

### Genotyping and linkage map generation

Isolation, sequencing, and genotype calls are fully described in [42]. Briefly, genomic DNA was extracted from caudal fin tissue, used to generate ddRADseq libraries, and sequenced on an Illumina HiSeq with 100bp paired end reads. Following demultiplexing and filtering of low-quality reads, reads were aligned to the *Metriaclima zebra* UMD2a reference genome. This genome was used as it is the best annotated Lake Malawi genome, and genomes were not available for either parental species used. This choice was not likely to result in misalignments as Lake Malawi species have >99.75% sequence similarity in pairwise comparisons [30]. However, we do note putative inversions on LG11 and LG20 between the genome of our hybrids and *M*. *zebra* [43]; this inversion may increase the size of the intervals mapped to these regions. Using the *M*. *zebra* reference genome, genotypes were called for those markers that had alternative alleles between the parents (i.e., AA x BB) and had a stack depth of 3. Additionally, a chi-square test was performed with the function geno.table to detect markers with distorted segregation patterns violating Hardy-Weinberg equilibrium; markers with a Bonferroni-corrected p-value < 0.05 were removed. As mentioned above, an inadvertent fertilization event led to two grandsires in this cross. To focus on species-level genetic contributions, markers were excluded if *Aulonocara* sires had discrepant genotypes or Hardy-Weinberg equilibrium was not met. Any phenotypic effects of genetic variation from a single grandsire (that is, intraspecies variation) is expected to be diluted in this cross and therefore not be identified in the subsequent QTL mapping described in the following section.

Generation of the linkage map is fully described in [42], was built in R (version 4.0.3) with the package R/qtl (version 1.44–9) [44], and used custom scripts available at https://github.com/kpowder/Biology2022. Briefly, RAD markers were initially binned into linkage groups according to their position in the *M*. *zebra* UMD2a reference genome, cross referenced based on segregation patterns and recombination frequencies, and removed if located in unplaced scaffolds that had more than 40% of missing data or did not demonstrate linkage disequilibrium with multiple markers in a single linkage group. Additional manual curation was used to minimize the number of crossovers for those markers whose recombination frequency profile did not match their position in the linkage map, likely due to being within a misassembled region of the reference genome or a structural variant. The final genetic map included 22 linkage groups, 1267 total markers, 19–127 markers per linkage group, and was 1307.2 cM in total size. The 22 linkage groups correspond to the 22 chromosome karyotype reported for both *Aulonocara* and *Metriaclima* genera [45], and markers that were removed based on the above criteria did not form additional linkage groups.

### Quantitative trait loci (QTL) analysis

QTL analysis used the R/qtl package (version 1.44–9) [46, 47] following [48]. Scripts are described in [49] and available at https://github.com/kpowder/MiMB_QTL. A multiple-QTL mapping (MQM) approach was used to more accurately identify intervals and their effects [48].The approach starts by using the onescan function in R/qtl [44] to identify putative, unlinked QTL. These putative QTL were used as cofactors to build a statistical model, and were verified by backward elimination to generate the final model. Statistical significance was assessed using 1000 permutations to identify 5% (significant) and 10% (suggestive) cutoffs. For each of these QTL peaks, 95% confidence intervals on each linkage group were identified by Bayes analysis. S2 Table includes for each trait the cofactors used to build models, significance cutoffs, confidence intervals, and allelic effects at the peak marker of the QTL interval.

### Identification of candidate genes

Markers are named based on physical locations (contig and nucleotide position) in the *Metriaclima zebra* UMD2a reference genome. These nucleotide positions were used in the NCBI genome data viewer (https://www.ncbi.nlm.nih.gov/genome/gdv, *M*. *zebra* annotation release 104) to retrieve candidate Entrez gene IDs and genomic locations. If the extremes of the 95% confidence interval included markers that mapped to unplaced scaffolds, the closest marker that mapped to a placed scaffold was used as an alternative. Full gene names were extracted from the Database for Visualization and Integrated Discovery (DAVID) [50, 51] using Entrez gene ID numbers as a query. Genes previously associated with body pigmentation or melanophore/melanocyte development were extracted from the annotated Molecular Signatures Database [52], which is used for Gene Set Enrichment Analysis [53]. A total of 258 genes from the human data set were used, which associated with the gene ontology (GO) cellular component term pigment granule (GO:0048770) and the following biological process terms: cellular pigmentation (GO:0033059), developmental pigmentation (GO:0048066), establishment of pigment granule localization (GO:0051905), melanocyte differentiation (GO:0030318), melanocyte proliferation (GO:0097325), melanosome assembly (GO:1903232), pigment accumulation (GO:0043476), pigment biosynthetic process (GO:0046148), pigment catabolic process (GO:0046149), pigment cell differentiation (GO:0050931), pigment granule localization (GO:0051875), pigment granule maturation (GO:0048757), pigment granule organization (GO:0048753), pigment metabolic process (GO:0042440), pigmentation (GO:0043473), positive regulation of developmental pigmentation (GO:0048087), positive regulation of melanocyte differentiation (GO:0045636), regulation of melanocyte differentiation (GO:0045634), regulation of pigment cell differentiation (GO:0050932), and regulation of pigmentation (GO:0120305).

## Results

### Phenotypic variation in barring

We sought to examine the genetic factors that control variation in vertical bars across the flank, particularly the levels of eumelanin produced by melanophores and the spacing of bars and interbars. To accomplish this, we generated a hybrid cross between two species with distinct barring patterns. Importantly, given that both parents demonstrate some degree of barring, this cross is unlikely to identify genomic regions that are master regulators of bars (i.e., presence versus absence of bars), but rather how bars can be modified when they are present.

**Fig 2:**
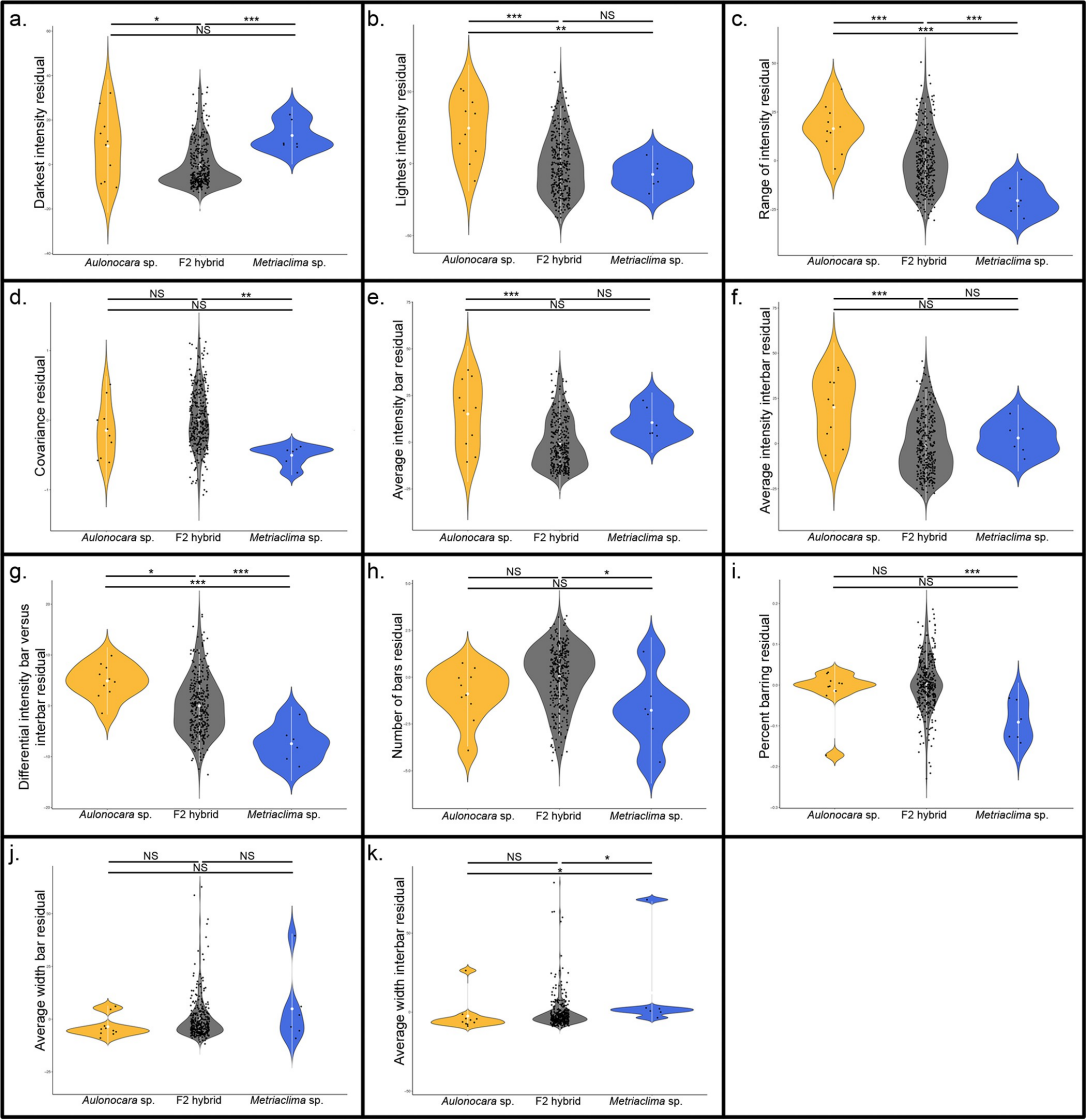
Variation in barring levels and patterns among *Aulonocara koningsi*, *Metriaclima mbenjii*, and their F_2_ hybrids. One set of measures relates to pigment levels produced by melanophores and are (a) darkest intensity, (b) lightest intensity, (c) range of intensity, (d) covariance, (e) average intensity of bars, (f) average intensity of interbars, and (g) differential intensity bars versus interbars. A second set of measures relates to the pattern of the bars and are (h) the number of bars, (i) percent barring, calculated as sum of total width of bars divided by total width of the isolated region, (j) average width of bars, and (k) average width of interbars. Significance in violin plots is based on ANOVA analysis followed by Tukeys HSD (data in S3 Table; p-values indicated by * <0.05, ** <0.01, *** <0.005).

*Aulonocara koningsi* are distinguished by a regular series of vertical, melanic bars that extend on the anterior-posterior axis from the opercle to the caudal peduncle, and throughout the dorsal-ventral axis (Fig 1A). Melanic bars in *Aulonocara koningsi* are broken up by pale, lightly pigmented interbars that tend be narrower or the same width as melanic bars (Fig 1A). Alternatively, *Metriaclima mbenjii* have vertical bars that typically occur on the flank only between the opercle and anal fin, then become more irregular or stop towards the posterior of the animal (Fig 1B). Interbars on *Metriaclima mbenjii* are more melanic, such that the overall effect of barring in this species is a subtle vertical bar on a dark background (Fig 1B). In agreement with these qualitative observations, quantification of the level of melanic pigmentation reveals that compared to *Metriaclima mbenjii*, black or brown pigment in *Aulonocara koningsi* is not significantly darker (Fig 2A, p = 0.62) but can be significantly lighter (Fig 2B, p = 0.0070). This generates a significantly larger range of pigment (Fig 2C, p = 5.90e-6) and larger differences between pigment levels in bars and interbars (Fig 2G, p = 5.20e-5).

Though they have a visual difference in barring patterns, we did not find that parental species were significantly different in their patterns of vertical bars (Fig 2G–2J, p = 0.052 to p = 0.53). The exception to this is that *Metriaclima mbenjii* had significantly wider interbars (Fig 2K, p = 0.02), though this is likely driven by the fact that the posterior of the flank in this species often did not have distinct bars, and due to a single specimen that had only a single bar and nearly the whole length of the body was classified as an interbar. We expect two factors explain the lack of statistical significance for most of the traits examined between the parental species. First, *Metriaclima mbenjii* is noted for a high percentage of ‘orange blotch’ (OB) animals, where melanophores are organized in irregular patches rather than bars. After removing these animals from the data set in order to focus on barring phenotypes, this only left six *Metriaclima mbenjii* parental specimens, reducing our statistical power. Second, our analysis that classified bars and interbars can have errors in classification of bars and interbars for an animal in which the grayscale intensity has less range and more inconsistent fluctuations, which was observed in many of the *Metriaclima mbenjii* parentals (i.e., compare pattern of the graph in Fig 1A versus variation around the average value in Fig 1B). An inability to accurately define bars and interbars in *Metriaclima mbenjii* parentals would influence measures of the average intensity of bars, the average intensity of interbars, the differential intensity between bars and interbars, the number of bars, the percent of barring, the average width of bars, and the average width of interbars, most of which did not show significant differences between parentals (Fig 2E–2K).

Despite this, it’s important to note that 100% of the 322 F_2_ hybrids demonstrated a distinct barring pattern, resembling the *Aulonocara koningsi* parental phenotype, and thus would have bars and interbars defined accurately by our analysis approach. This observation agrees with a previous suggestion that several genes are sufficient to drive the formation of bars [29]. Though they all had melanic vertical bars, F_2_ hybrids were phenotypically varied in terms of the level of eumelanin produced in the bars and interbars, as well as the spacing of these pigment elements. This population therefore can yield valuable insights into the patterns of phenotypic variation in barring, as well as the genetic loci that regulate this.

Color differences between males and females is thought to be sexually-selected, resulting in widespread sexual dimorphism across vertebrates [4, 54–56], including cichlids [16, 17, 34, 57]. Within this cross however, we find no statistical relationship between any measures of pigment level or patterns and sex (p = 0.24 to 0.89, S3 Table), noting that F_2_ animals were collected as juveniles and did not express fully mature nuptial coloration. Thus, variation identified here reflects differences due to species-specific genetic polymorphisms. Notably, within Lake Malawi cichlids, a set of ancestral polymorphisms are being recombined in differing combinations among species [30, 58, 59]. Thus, even for traits with non-significant differences between parental species, QTL mapping can identify genetic factors that underlie pigment variation within this radiation and genetic combinations that are possible in other species.

### Genetic basis of variation in pigment phenotypes

**Fig 3:**
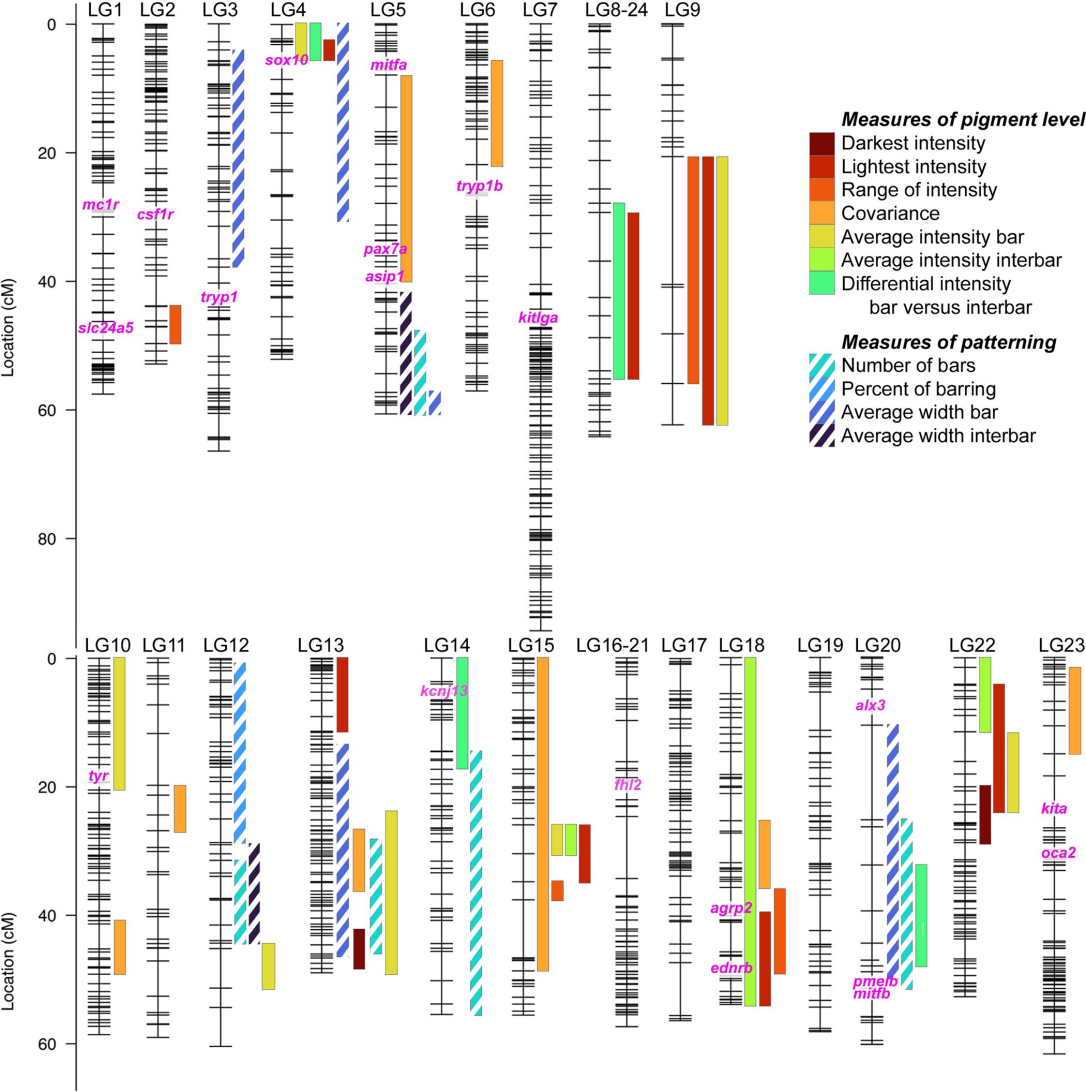
Quantitative trait loci (QTL) mapping identifies 48 intervals associated with variation in barring between *Metriaclima mbenjii* and *Aulonocara koningsi*. Each linkage group (LG, i.e., chromosome) has markers indicated by hash marks. Bar widths indicate 95% confidence interval for each QTL and bar color indicates the pigment trait analyzed. Candidate genes previously associated with variation in eumelanin production and development of stripes or bars (see main text for references) are in pink text, with their genomic locations indicated on linkage groups. Additional candidate genes *pax3a* and *pmela* are located in unplaced scaffolds in the *M*. *zebra* UMD2a reference genome and not included here. Illustrations of each trait are in Fig 1. QTL scans at the genome and linkage group level are in S3 and S4 Figs, respectively. Details of the QTL scan, including statistical model and physical locations defining each QTL are in S2 Table.

To determine the genetic basis of variation in barring phenotypes, we genetically mapped seven traits related to the level of eumelanin produced and four traits related to the location of bars. Fifty-one QTL underlie quantitative differences in pigmentation in *Metriaclima* x *Aulonocara* F_2_ hybrids, with 48 that reach 5% statistical significance at the genome-wide level (Fig 3, S3 and S4 Figs, and S2 Table). An additional 3 QTL are suggestive at the 10% level and included in supplemental material only (S3 and S4 Figs and S2 Table). QTL are found on 17 of 22 linkage groups, with each linkage group containing 1–6 significant loci each. Interestingly, four of our QTL overlap with regions previously associated with the presence or absence of barring [29]: the QTL on LG5 for covariance, the QTL on LG13 for lightest intensity, and two QTL on LG14 for the differential intensity of bars versus interbars and the number of bars. While more work is needed to fine map these QTL, this leaves open the possibility that a single gene could regulate whether an animal has bars as well as the expression of this trait.

Between one and six QTL contribute to each trait, with each interval explaining 4.75–16.1% of variation for each trait (average 7.25% variation explained, S4 Fig and S2 Table). While these data indicate that all these pigment traits are multifactorial, the QTL identified cumulatively explain a considerable portion of variation for multiple traits. Five QTL together explain 42.66% of variation in the number of bars, eight QTL explain 44.07% of variation in the average intensity of bars, seven QTL combine to explain 50.61% of variation in lightest intensity, and eight QTL cumulatively explain 69.14% of the total variation in covariance, or the discrepancy between dark bars on a lightly pigmented flank.

There is a large degree of overlap in our QTL intervals, which is expected given both the traits analyzed and their degree of correlation. For example, LG9 contains three QTL that overlap between 20.7–56.0 cM and control the lightness intensity, range of intensity, and average intensity of bars (Fig 3 and S2 Table). A change in lightness would directly affect the calculation of the range of intensity, explaining why these traits map to the same interval. Accordingly, these traits are highly correlated (r = 0.90, p < 2.2e-16, S4 Table) and the lightest intensity is also correlated with the average intensity of bars (r = 0.87, p < 2.2e-16, S4 Table). These types of phenotypic correlations also explain the overlapping QTL on LG15 from 26.0–30.5 cM for lightest intensity, average intensity of bars, and average intensity of interbars (Fig 3 and S2 Table), traits that all correlate (r = 0.87 to 0.96, p < 2.2e-16 for all pairwise correlations, S4 Table). However, it’s important to note that even highly correlated traits can be regulated through distinct mechanisms (i.e., many-to-one mapping [60]). For example, darkest intensity, average intensity of bars, and average intensity of interbars are all highly correlated (r = 0.84 to 0.95, p < 2.2e-16 for all pairwise correlations, S4 Table). Together, these traits map to fourteen intervals, but depite a high correlation of the phenotypes, ten of these QTL are unique genomic regions. Further, five of the eight QTL for the average intensity of bars are on LGs that do not contain any QTL for the other two correlated traits.

The effect of specific alleles on phenotypes is varied across phenotypes (S4 Fig and S2 Table). For instance, for the QTL on LG12, the allele inherited from the *Metriaclima mbenjii* granddam decreases the number of bars on LG12, but increases the number of bars on LG13. Three other examples highlight the complex genetic interactions that are possible within these species and across the cichlid radiation. First are the set of overlapping QTL on LG9 and cluster of QTL on LG22. In both cases, alleles inherited from *Metriaclima mbenjii* are associated with lighter intensity values (S4 Fig and S2 Table), though lighter pigmentation is associated with the *Aulonocara koningsi* phenotype (Figs 1A, 1B and 2B). The second example is a group of QTL related to pigmentation levels that occur on LG15. All 5 QTL demonstrate an underdominant inheritance pattern, such that a combination of heterozygous alleles explain the variation in lightest intensity, range of intensity, covariance, average intensity of bars, and average intensity of interbars (S4 Fig and S2 Table). These suggest that complex interactions between genes (i.e., epistasis [61, 62]) regulate these phenotypic traits such that the effects of the *Metriaclima mbenjii* allele are only visible in the absence of or in combination with additional alleles. These examples of cryptic genetic variation [63, 64] are important to understand the full spectrum of genetic factors that regulate a complex trait like pigmentation, and allelic combinations that are likely to be present in other cichlid species within Lake Malawi [30, 58, 59].

### Distinct genetic origins underlie pigment levels and patterning

We hypothesized that a separate set of genetic, molecular, and developmental mechanisms may underlie variation in the level of melanic pigmentation and the patterns of vertical bars. That is, we predicted that one set of genes would control where melanophores would be located and capable of generating dark vertical bars (i.e., patterning), and a separate set of genes would then control how much eumelanin is produced by these melanophores (i.e., pigment level). One set of data supporting this is an analysis of correlations among phenotypic traits (S4 Table). While some measures of pigment levels are correlated as discussed above, none of the four measures related to patterning were strongly correlated with any of the seven measures of pigment level (r = -0.39 to 0.31 in pairwise comparisons, S4 Table). We also note differing degrees of significance of these correlations (S4 Table). That is, traits within a group (e.g., both traits related to pigment level) are more highly significant (p < 2.2e-16 to 0.11, average p = 0.0041) than the significance is of correlations between groups (e.g., one trait is pigment level and another is patterning, p = 4e-13 to 0.99, average p = 0.148) (S4 Table).

QTL data also largely supports that divergent genetic factors regulate pigment intensity and bar/interbar locations, through the examination of the degree of overlap of QTL intervals (Fig 3). We found numerous overlaps within similar types of traits. That is, LG5 and LG12 contain regions that have 2–3 overlapping QTL related to patterning, and LG8-24, LG9, LG15, LG18, and LG22 have 2–4 QTL at the same genomic locus that control levels of pigment produced.

**Fig 4:**
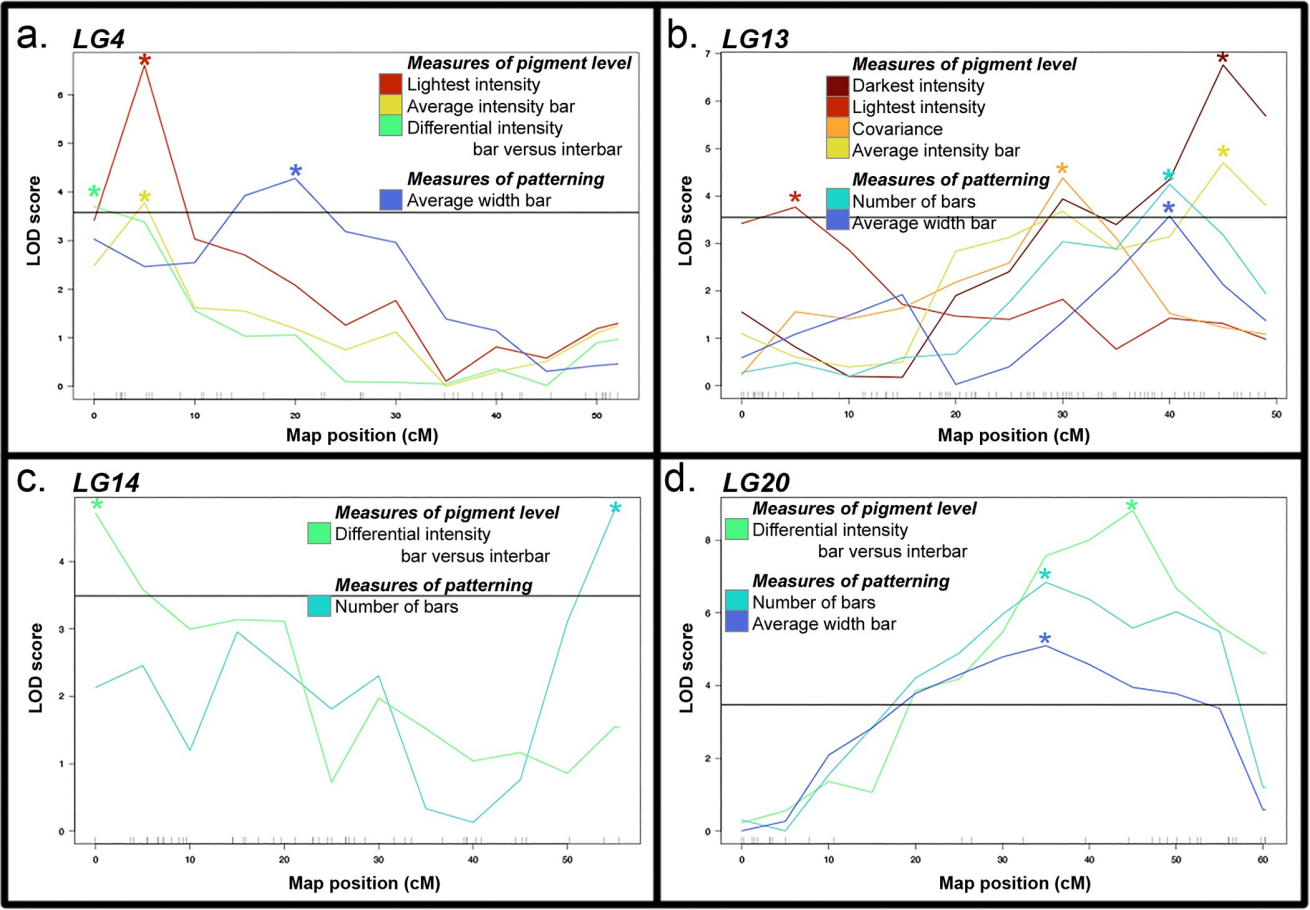
Quantitative trait loci (QTL) that underlie variation in pigment levels and pigment patterning are largely distinct. Included are all linkage groups—(a) LG4, (b) LG13, (c) LG14, and (d) LG20—in which QTL for pigment level and patterning have overlapping 95% confidence intervals as visualized in Fig 3. Colors represent trait, as indicated by the legend and as illustrated in Fig 1. Peak markers for each QTL are indicated by an asterisk in a color matching the trait. The solid horizontal line in each panel represents 5% significance, measured as the average value from each of the featured scans on that specific linkage group; averaging this significance did not cause any of these QTL to change from significant to non-significant or vice versa. Further details of the QTL are in S4 Fig and S2 Table.

In contrast, only LG4, LG13, LG14, and LG20 have overlapping 95% confidence intervals for traits related to pigment level and patterning, and additional examination of these regions suggest that a potential pleiotropic effect on both aspects of pigmentation is limited to LG13 and LG20 (Fig 4). For instance, all four QTL on LG4 have 95% confidence intervals that overlaps from 0–5.79 cM (Fig 3 and S2 Table). However, this includes the peak and full region for the three traits related to pigment level, while the QTL for patterning has a peak at 20 cM and dips below significance under the peak of the other QTL, suggesting that two linked, but distinct loci may underlie variation in these traits (Fig 4). Another overlap occurs on LG14, where a QTL for the differential intensity between bars and interbars overlaps with a QTL for the number of bars from 14.6–17.32 cM (Fig 3 and S2 Table). However, closer examination of these loci reveals that the peak for these QTL are on opposite ends of the linkage group, 55 cM apart (Fig 4), and this overlapping interval is unlikely to contain a causative gene that underlies both the pigment level phenotype and the patterning phenotype.

However, two regions may regulate both pigment intensity and location on the body. First is on LG20, where a QTL for differential intensity between bars and interbars resides within QTL for number of bars and average width of bars (Fig 3). While the peak for the patterning QTL are both at 35 cM and the peak for the pigment level QTL is at 45 cM, these three all feature broad peaks with a high degree of overlap (Fig 4 and S2 Table). Finally, six separate QTL reside on LG13 and span the entirety of the chromosome (Fig 3). Examination of the peaks and confidence intervals (Figs 3 and 4 and S2 Table) suggests that at least 3 separate regions of LG13 are contributing to the pigment traits measured. This includes a region from 0–11.37 cM containing a QTL for lightest intensity, a region from 26.11–36.24 cM with a QTL for covariance, and a region from 42.85–48.33 cM that includes a QTL for darkest intensity (Figs 3 and 4). These three QTL do not overlap each other, but the latter two both overlap QTL for average intensity of bars, number of bars, and average width of bar (Figs 3 and 4). Thus, in the case of both LG13 and LG20, more detailed mapping will be necessary to determine if these traits are regulated by the same gene, distinct genes that are in close physical distance or linkage with each other, or distinct genomic loci.

## Discussion

Though it is one of the most common pigment patterns in fishes, we know relatively little about the genetic basis of dark vertical barring [16]. To address this, we used a genetic mapping cross between two cichlids with distinct presentations of barring and mapped 48 genetic loci that influence the relative levels of melanic pigment in bars and interbars, as well as their patterning. In addition to identifying this series of quantitative trait loci, we found eumelanin pigment levels and barring patterns are largely regulated by independent loci. These separate, polygenic genetic architectures would enable evolutionary fine-tuning of barring in response to natural or sexual selection, promoting further diversity in pigmentation. Further, we observed that vertical barring is not due to a master regulatory gene, but a combination of genetic factors. This directly contrasts with two other traits in cichlids with distinct arrangements of melanophores, horizontal stripes and blotching, controlled by *agouti-related protein 2* (*agrp2*) and *paired box 7a* (*pax7a*), respectively [19, 21]. Our data thus supports a previous suggestion that barring in cichlids is polygenic [29].

Our loci add to a variety of genetic factors of both small and large effect that regulate pigmentation in cichlids [21, 28]. These alleles for fin pigmentation [22–24, 28], xanthophore-based red and yellow coloration [26, 28], melanophore-based black and brown coloration [19, 21, 28], and integration versus modularity of color patterns across the flank [28] are shuffled in differing combinations [30, 58, 59] to generate the range of colors and patterns that characterize the adaptive radiation of cichlids [16, 17, 65]. These pigmentation patterns, whether inherited independently or not, are then subject to a variety of ecologically-relevant selective pressures such as predator avoidance and intrasexual competitive interactions [4, 6–13]. One critical implication of these hues and patterns is assortative mating [66, 67], which can directly result in reproductive isolation, and thus sexual selection, which is central to the dramatic speciation and divergence of cichlids [14, 18, 68, 69].

The majority of our QTL do not include a series of genes previously associated with variation in pigment (Fig 3) in cichlids [19, 21–23, 26–28], *Danio* species including those with vertical barring [70–75], cavefish [76, 77], sticklebacks [78], and other non-fish vertebrates [5, 75, 79–82]. For instance, *melanocortin 1 receptor* (*mc1r*) on LG1 has been associated with a series of adaptive pigment changes, through regulation of the biosynthesis of eumelanin [5]. While activating or repressing eumelanin production would likely influence any of the seven traits related to pigment level that we measured, none of the 35 QTL that underlie these traits include *mc1r*. Additional work will be needed to narrow genetic intervals, verify candidate genes, and identify the molecular and cellular mechanisms that generate the phenotypes we mapped. Most of our genetic intervals contain many genes, from 40 genes for covariance on LG10 to the entire chromosome and 1069 genes for the average intensity of interbars on LG18 (average = 342 genes, S5 Table). However, we discuss below a number of strong candidate genes within these intervals and how they may mediate variation in melanic traits.

One set of candidate genes are associated with the development and survival of the melanophores themselves, including the trunk neural crest cells that are the embryonic source of these pigment cells [7, 8]. Variation in the induction, migration, and differentiation process could change the number and/or location of melanophores present within the skin to generate changes in both the pattern of barring and the intensity of melanin produced. For instance, a QTL for average width of bars on LG4 includes the candidate gene *SRY-box 10* (*sox10*). We note that *sox10* is near, but not included in QTL for the lightest intensity, average intensity of bars, and differential intensity between bars and interbars that partially overlap this QTL for average width of bars (Fig 3 and S2 Table). *Sox10* is necessary for neural crest cell specification [83, 84] and required to establish the melanophore linage [85]. Genetic variation in this gene in humans results in Waardenburg syndrome, which is characterized by a suite of alterations to neural crest cell derivatives, one of which is depigmented patches in the skin and hair [86, 87]. This is not the only candidate gene associated with a pigmentation condition in humans. Overlapping QTL on LG13 contribute to covariance, the number of bars, and the average width of bars (Fig 3). These three QTL all include the candidate gene *F-box protein 11a* (*fbxo11a*) and the QTL for number of bars and average width of bars also include *SPARC-related modular calcium binding 2* (*smoc2*) (S5 Table). Both genes are associated with the human condition vitiligo, characterized by the progressive loss of pigment cells through cell death or autoimmunity [88–91]. Though little is known about the molecular function of *smoc2*, *fbxo11* regulates melanophore proliferation, apoptosis, and intracellular transport of the eumelanin biosynthesis enzyme tyrosinase [92]. Such a loss of melanophores in localized regions could result in the changes in barring pattern or amount of melanin produced and counting of individual melanophores [25, 41] may provide further insights into this regulation.

The location and number of melanocytes would also be impacted by genes that regulate fate decisions during melanophore development. The QTL for covariance on LG5 includes the gene *pax7a*, and a related gene, *paired box 3b* (*pax3b*), is located within a QTL on LG14 for differential intensity of bars and interbars. *Pax3a*, the paralog of our candidate, and *pax7a* and can act transcriptionally as switch factors between different pigment cell fates, and these genes have previously been associated in cichlids with changes in the balance of melanophore and xanthophore cell numbers, changes in pigment levels, or altered patterns such as melanic blotches [19, 20, 28, 93]. An overlapping QTL for the lightest intensity and the average intensity of interbars on LG18 includes another candidate gene related to cell fate decisions. *Endothelin receptor type B* (*ednrb*) is required for differentiation of melanophores [94] and another pigment cell in fishes, iridophores [95]. Mutations in *ednrb* result in broken stripes in zebrafish [70], though it has yet to be determined if the spots caused by this fate switch could merge into a bar pattern instead of stripes. Importantly, the allelic effects (S2 Table and S4 Fig) for LG18 for the lightest intensity match the trend seen in parentals (Fig 2B), with the *Aulonocara* allele causing a lighter intensity in an additive way. Also within this interval on LG18 is the master switch for horizontal stripes in cichlids, *agrp2* (Fig 3). Previous work has predicted that stripes and bars are regulated by genetically-independent modules [29], suggesting that *agrp2* is not the causative gene on LG18 for changes in our bar phenotypes. However, it is possible that this independence depends on the cichlid species being compared, and *agrp2* may regulate barring in *Metriaclima* and *Aulonocara*.

Once melanophores are specified and migrate to their position on the flank, variation in eumelanin biosynthesis can produce variation. This would be expected to change pigment intensity, but not the pattern of barring. In agreement with this, the three QTL intervals described below that contain candidate genes associated with eumelanin production are associated with at least one of the measures of pigment level, but none of the measures of bar location. The LG5 QTL for covariance and the LG10 QTL for average intensity of bars contain two genes that have been associated with the biosynthesis of melanin across multiple vertebrate species, *agouti signaling protein* (*asip*) and *tyrosinase* (*tyr*) [5]. Interestingly, *asip* is also associated with countershading, a dorsoventral gradient of pigmentation important for predator avoidance, the switch between production of dark eumelanin and yellow/red pheomelanin, and may also repress melanophore differentiation [96–98]. Candidate genes may also regulate melanin production based on ecological triggers. For instance, within the LG15 QTL associated with lightest intensity, covariance, average intensity of bars, and average intensity of interbars is the candidate gene *melanocortin 2 receptor accessory protein 2* (*mrpa2*). This gene is involved in the melanocortin response pathway, and can modulate melanin levels following starvation- or crowding-induced stress [99].

Finally, variation in barring can be generated by differences in pigment density and distribution or alteration of melanophore cell density and shape [32]. While this can be rapidly regulated in cichlids through physiological changes such as hormone signaling [41, 100], this can also be regulated at the genetic level. Within the overlapping regions on LG4 for QTL regulating lightest intensity, average intensity of bars, and differential intensity between bars and interbars is *melanin-concentrating hormone receptor 1b* (*mchr1b*). This G-protein coupled receptor integrates with the nervous system to regulate hormonal changes in pigment aggregation as a fish alters its pigmentation for camouflage from predators, to attract a mate, or in response to intrasexual competition [101, 102]. For both lightest intensity and differential intensity, the inheritance of an allele from the *Aulonocara* grandparent results in a lighter pigmentation or an increase differential in pigmentation in a dominant way (S2 Table and S4 Fig), matching the observation in parentals of *Aulonocara* having significantly lighter interbars to contrast with the dark bars (Figs 1A, 1B and 2B). Note that we are unable to evaluate if the allelic effects match the trend of the parental phenotypes for the average intensity of bars as this is not significantly different between parentals based on our method of quantification (Fig 2E, and see Results for further explanation). Another strong candidate gene is found within a QTL on LG14 for differential intensity between bars and interbars. *Potassium inwardly rectifying channel subfamily J member 13* (*kcnj13*) is necessary for interactions between melanophores and other pigment cell types such as iridophores and xanthophores, resulting in localized changes in chromatophore shape and changes in the contrast of pigment patterns [103]. Notably, *kcnj13* is associated with the evolution of vertical barring in *Danio* species, suggesting a conserved role in barring across a large portion of the fish phylogeny [73]. Finally, a QTL for the number of bars on LG20 includes the gene *premelanosome protein b* (*pmelb*). *Pmelb* encodes a protein specific to pigment cells, that affects the cellular structure and shape of melanosomes through formation of fibrillar sheets on which melanin polymerizes and is deposited [104–106]. Further, CRISPR inactivation of paralogs *pmela* and *pmelb* in tilapia resulted in a reduction of melanophore number and size, as well as a loss of a vertical barring pattern [26].

Further work is needed to determine if the candidate genes described above are those driving variation in barring pattern, or if other genes in the intervals (S5 Table) with yet unknown roles in melanophore biology contribute to variation in these traits. Additional research is also needed to define the evolutionary forces that drive variation in pigmentation. Within cichlids, pigmentation has been proposed to be driven by sexual selection including mate choice and male-male competitive interactions [18, 33, 34, 68]. However, the evolution of barring may be driven by natural selection based on camouflage and predator avoidance [6, 31, 32] or pleiotropic effects of alleles under selection for traits associated with habitat or trophic specialization. For example, the QTL for the average width of interbars on LG12 includes *ptch1*, which has been associated with the shape of the lower jaw and feeding mechanics in cichlids [107]. We also find overlapping QTL for both pigmentation and body shape in this cross. Specifically, QTL for the average width of barring and body depth overlap from 27.6–37.8 cM on LG3 and QTL for anal fin length, the range of pigment intensity, lightest melanic pigment intensity, and the average intensity of bars overlap from 20.7–56.0 cM on LG9 [42]. While a more thorough analysis of the genetic hotspots driving cichlid evolution is needed, these observations suggest that multiple aspects of cichlid divergence may be under the control of pleiotropic alleles.

## Conclusions

Pigment hues and patterns can be selected by a series of natural and sexual selective pressures including predator avoidance, mate choice, and competitive interactions. Here we explore one common pattern with the diverse coloration found in cichlid fishes, vertical melanic barring, for which the genetic and molecular basis is largely unexplored. We show here that the genomic intervals that influence pigment levels are largely distinct from those that regulate bar patterning, which can promote the degree of variation that is possible in this trait. A series of candidate genes within these intervals highlight the varied ways that melanophore development can be altered to produce ecologically-relevant variation in barring. The pigmentation patterns studied here are particularly important for the adaptive radiation of cichlids, where they play a role in sexual selection and reproductive isolation, and therefore in maintaining species boundaries. Future studies identifying the causative alleles for the QTL we identify here will allow exploration of their evolutionary history across the cichlid radiation, and their potential role in speciation.

## Supporting information

S1 Fig Color images of representative parental and hybrid animals. Animals were euthanized in a cold solution to relax chromatophores and maximize the visible eumelanin-based pigmentation from melanophores. Photographs were taken using standard lighting conditions and color-balanced using a gray scale color standard.
(TIFF)

S2 Fig Determination of thresholds used to define bars. Empirical assessment was used to identify the appropriate gray intensity value to use as a cutoff to define bars and interbars. This was conducted on a test set of four *Aulonocara* parentals, four *Metriaclima* parentals, and four randomly-chosen hybrids, with two of each group visualized here. Each panel includes the picture of the entire animal on top and the isolated region used for analysis of barring on the bottom. Pixels in red are those in which the pixel is equal to or less than (i.e., darker than) the indicated intensity, and are considered within a bar. Pixels with a gray intensity value greater (i.e., lighter) than the indicated cutoff is considered within an interbar. The average intensity measure represents the average value of the isolated region, calculated for each individual specimen. Note that using a cutoff of the average intensity for each individual most accurately represents the pattern of barring observed by eye.
(TIFF)

S3 Fig Genome-wide QTL scans for pigment traits. Pigment traits analyzed are residual data for (a) darkest intensity, (b) lightest intensity, (c) range of intensity, (d) covariance, (e) average intensity of bars, (f) average intensity of interbars, (g) differential intensity bars versus interbars, (h) number of bars, (i) percent barring, calculated as sum of total width of bars divided by total width of the isolated region, (j) average width of bars, and (k) average width of interbars. Colors of the scan match colors used in Figs 1 and 3. Significance is indicated at the 5% (solid line) and 10% (dashed line) level. Details of QTL scans are in S2 Table. QTL scans by chromosome are in S4 Fig.
(PDF)

S4 Fig QTL scans by linkage group and allelic effects. Pigment traits analyzed are residual data for (a) darkest intensity, (b) lightest intensity, (c) range of intensity, (d) covariance, (e) average intensity of bars, (f) average intensity of interbars, (g) differential intensity bars versus interbars, (h) number of bars, (i) percent barring, calculated as sum of total width of bars divided by total width of the isolated region, (j) average width of bars, and (k) average width of interbars. 95% confidence interval for QTL is indicated by shading, percent of total phenotypic variation explained by QTL is reported, and genome-wide significance is shown at the 5% (solid line) and 10% (dashed line) level. Details of QTL scan are in S2 Table and genome-wide visuals are in S3 Fig. Allelic effects are shown for marker at the peak log odds (LOD) score for the QTL, which is indicated by *. The A allele was inherited from the *Metriaclima* granddam and the B allele from the *Aulonocara* grandsire.
(PDF)

S1 Table Body length and mass information for parental and hybrid populations. Mass was measured immediately upon sacrifice. As described in methods, the standard length (distance from tip of snout to the caudal peduncle) was measured as number of pixels from images and then converted to cm using a ruler in the image. Mean values and standard deviations are reported. Statistical comparisons are based on ANOVA analysis, followed by Tukeys HSD test.
(PDF)

S2 Table Details of quantitative trait loci (QTL) mapping and regions. For each QTL, we include cofactors use to generate models as well as markers and physical positions for the peak of the QTL and the 95% confidence interval. Marker names include the physical location on the linkage group, with names referring to the contig and nucleotide position in the *M*. *zebra* UMD2a assembly. LOD values, percent phenotypic variance explained by that QTL, allelic effects, and additive, dominance, and heritability calculations are for the peak marker in the QTL. QTL listed in gray are suggestive at the 10% significance level, while those in black meet 5% genome-wide significance based on values indicated.
(XLSX)

S3 Table Statistical significance of pigment measures. Effects of size (standard length) and sex were assessed by ANOVA analysis. Note that sex was only called for F_2_ hybrids, but not parental species. Significance between parental and hybrid groups on size-corrected (i.e., residual values) were assessed by ANOVA followed by Tukeys HSD. P-values < 0.05 are in bold red text.
(XLSX)

S4 Table Correlations between pigment traits. Calculations are for F_2_ hybrids animals only and exclude parentals. Except for correlations with standard length, all measures are size corrected prior to the calculation of correlation. Correlations with a p-value <1e-9 are in bold red text.
(XLSX)

S5 Table Candidate genes for pigment QTL. For the indicated 95% confidence interval, we include candidate gene names in the *M*. *zebra* annotation release 104, NCBI gene ID number, and physical positions in the *M*. *zebra* genome UMD2a assembly. Those genes that have been previously associated with pigmentation or melanophore development or function in the Molecular Signatures Database/Gene Set Enrichment Analysis (see methods for details) are noted. LOD scores are noted for genes near a genetic marker in the linkage map, but omitted for genes that fall between markers. Purple shading in the LOD score column is a heat map for LOD scores, from dark purple at scores close to the peak LOD score for the QTL to white for those scores furthest from the peak LOD score.
(XLSX)
