# Supplementary figures and images for: Distinct genetic origins of eumelanin levels and barring patterns in cichlid fishes
Source: PLoS One. 2024 Jul 8;19(7):e0306614. doi: 10.1371/journal.pone.0306614 (PMC11230561; doi:10.1371/journal.pone.0306614)

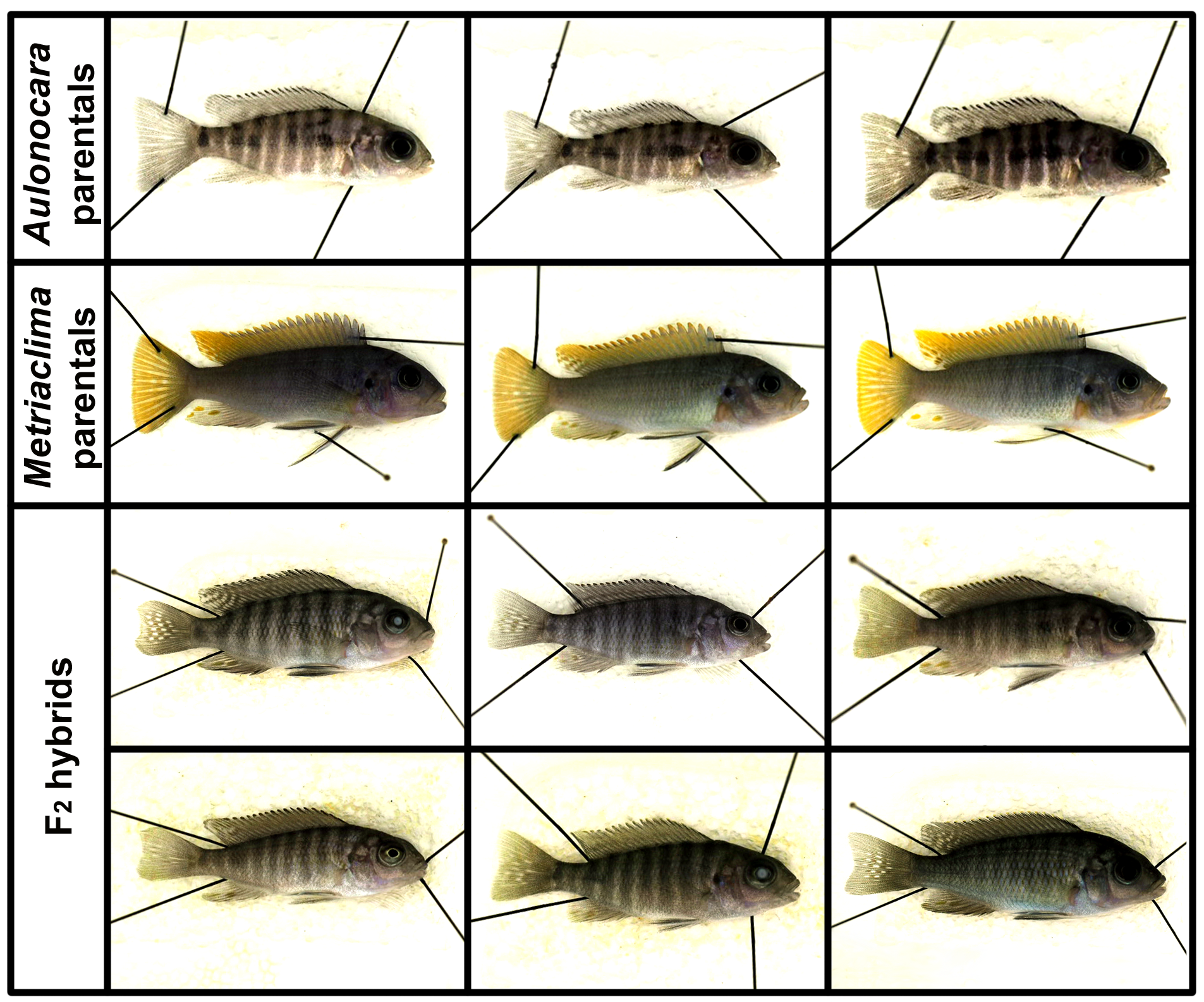

Supplement: S1 Fig — Animals were euthanized in a cold solution to relax chromatophores and maximize the visible eumelanin-based pigmentation from melanophores. Photographs were taken using standard lighting conditions and color-balanced using a gray scale color standard. (TIFF) [file pone.0306614.s001.tiff]

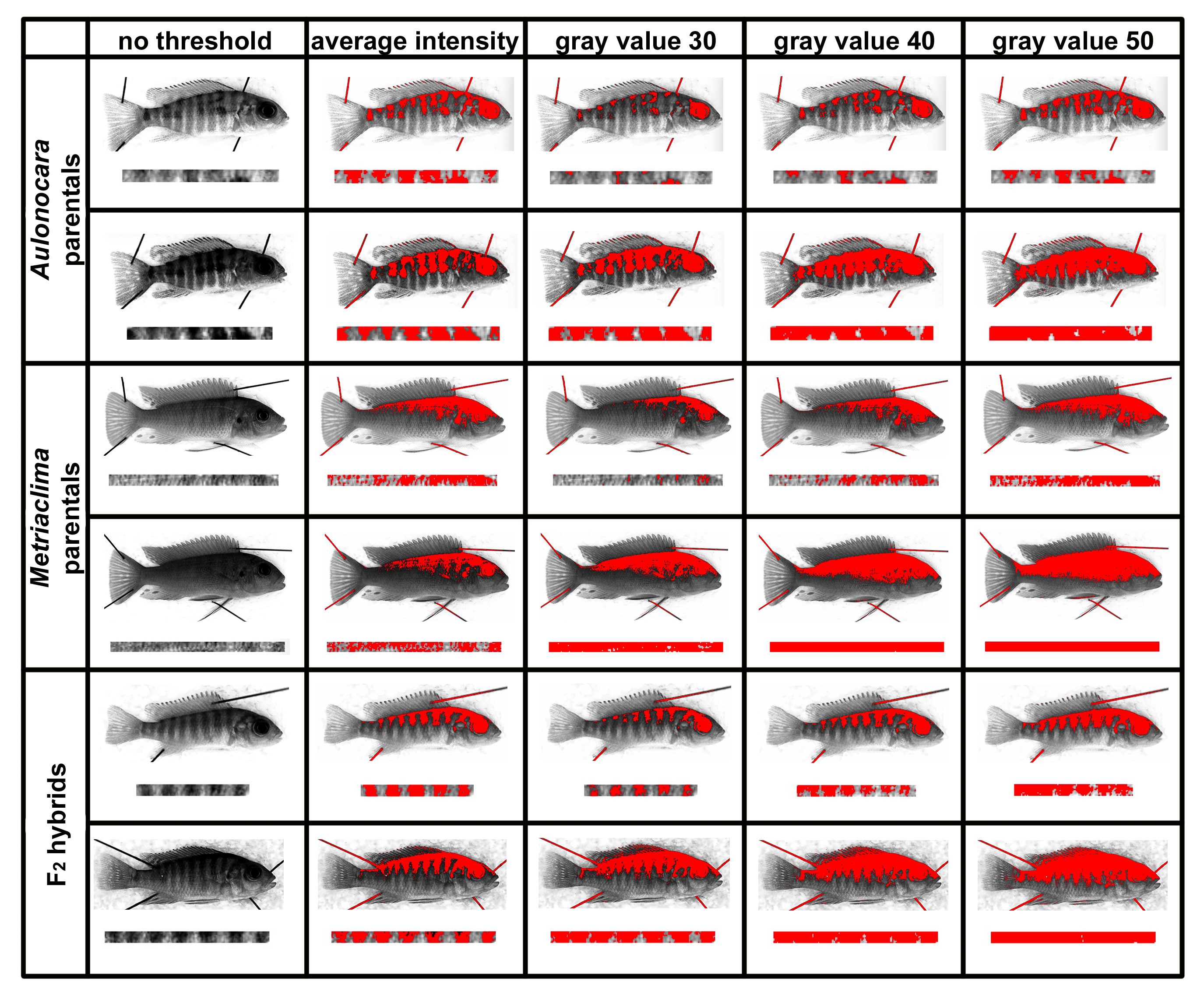

Supplement: S2 Fig — Empirical assessment was used to identify the appropriate gray intensity value to use as a cutoff to define bars and interbars. This was conducted on a test set of four Aulonocara parentals, four Metriaclima parentals, and four randomly-chosen hybrids, with two of each group visualized here. Each panel includes the picture of the entire animal on top and the isolated region used for analysis of barring on the bottom. Pixels in red are those in which the pixel is equal to or less than (i.e., darker than) the indicated intensity, and are considered within a bar. Pixels with a gray intensity value greater (i.e., lighter) than the indicated cutoff is considered within an interbar. The average intensity measure represents the average value of the isolated region, calculated for each individual specimen. Note that using a cutoff of the average intensity for each individual most accurately represents the pattern of barring observed by eye. (TIFF) [file pone.0306614.s002.tiff]

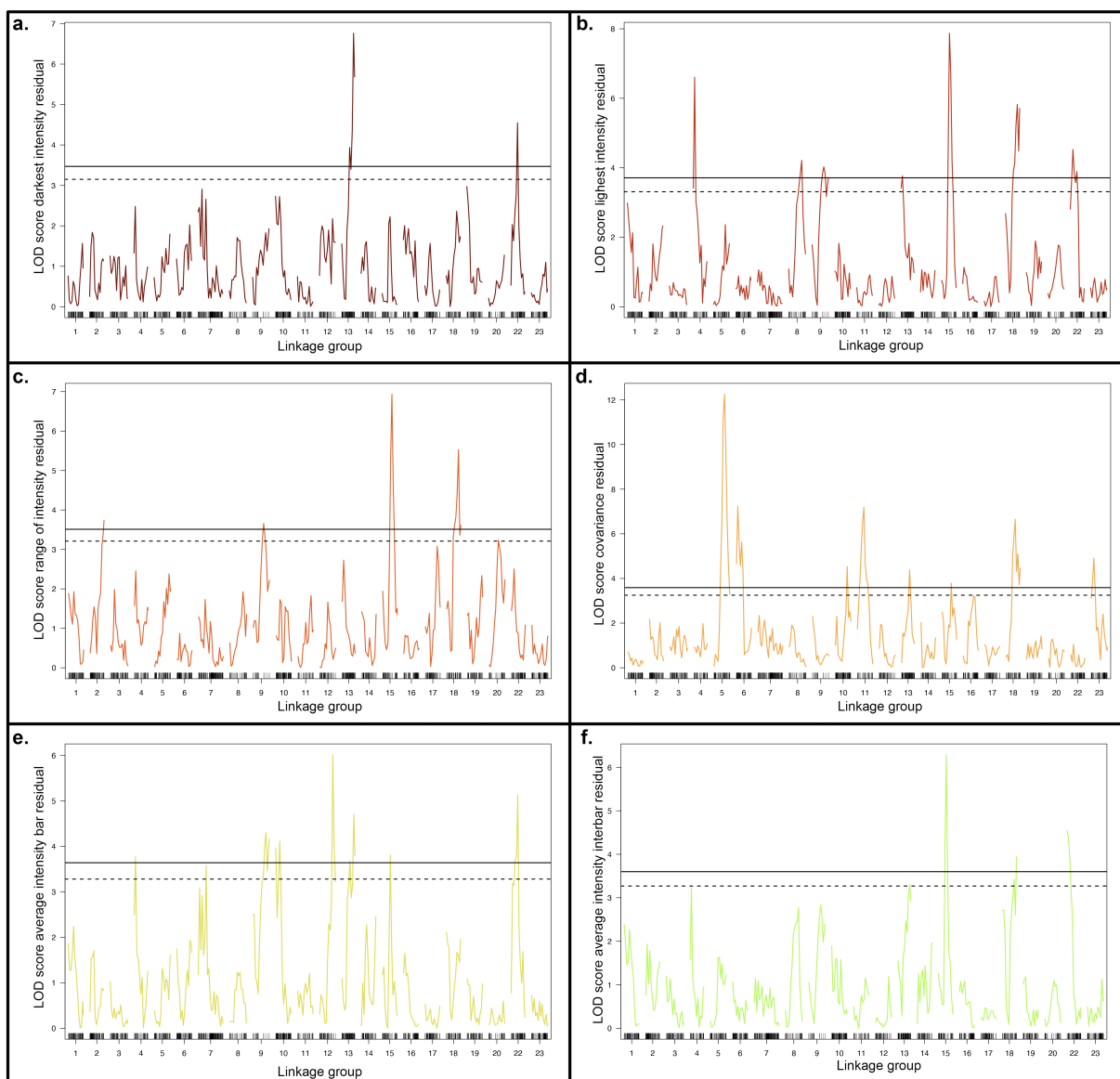

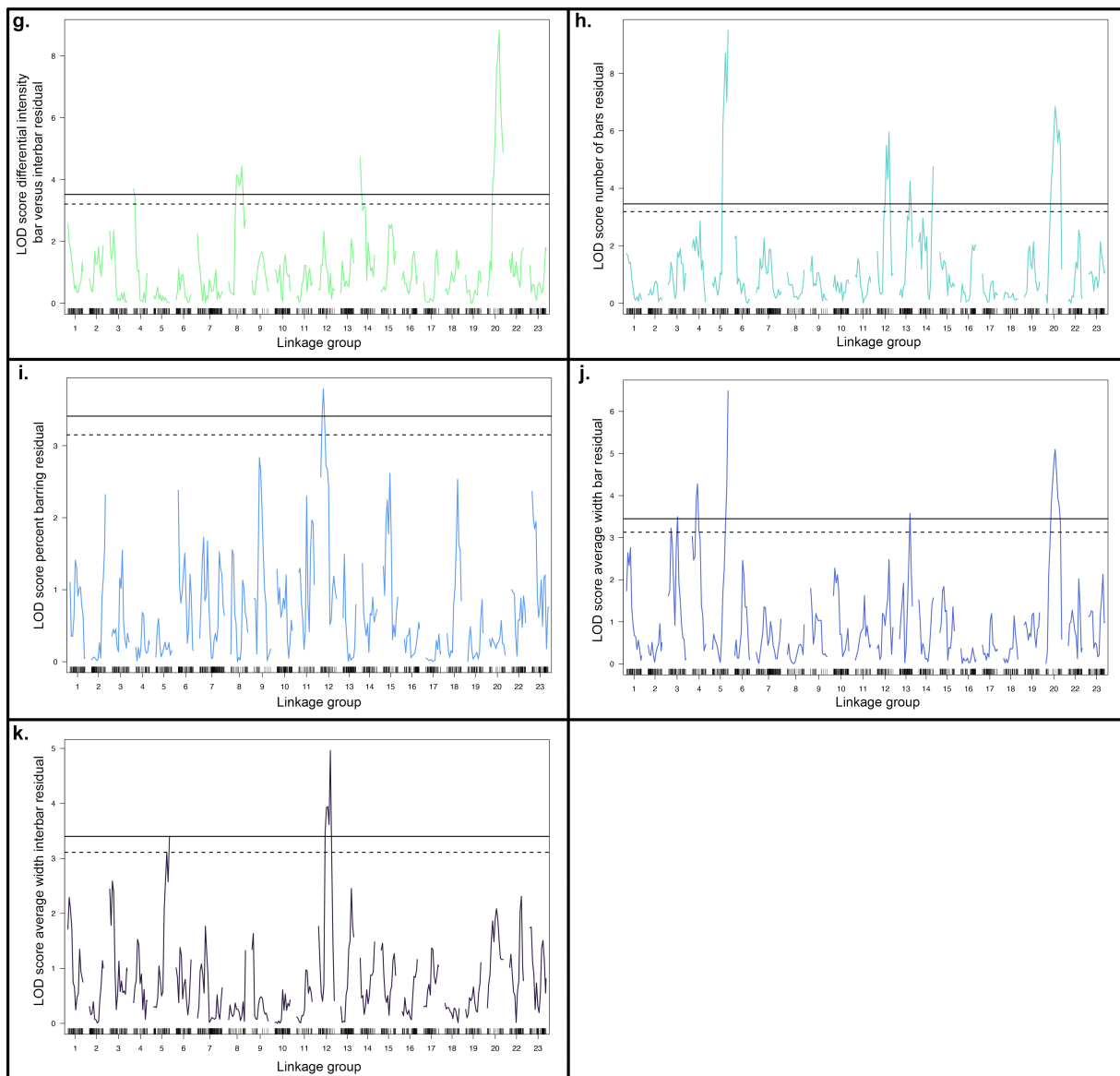

Supplement: S3 Fig — Pigment traits analyzed are residual data for (a) darkest intensity, (b) lightest intensity, (c) range of intensity, (d) covariance, (e) average intensity of bars, (f) average intensity of interbars, (g) differential intensity bars versus interbars, (h) number of bars, (i) percent barring, calculated as sum of total width of bars divided by total width of the isolated region, (j) average width of bars, and (k) average width of interbars. Colors of the scan match colors used in Figs 1 and 3. Significance is indicated at the 5% (solid line) and 10% (dashed line) level. Details of QTL scans are in S2 Table. QTL scans by chromosome are in S4 Fig. (PDF) [file pone.0306614.s003.pdf]

a.

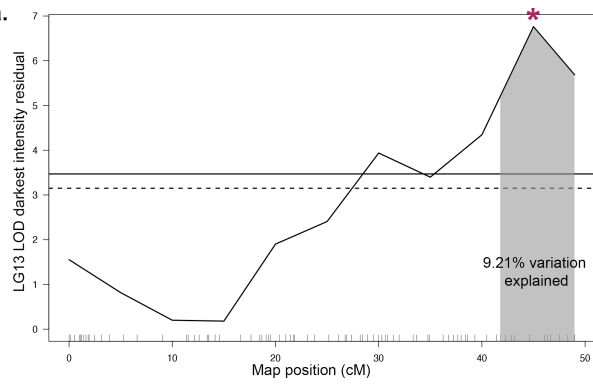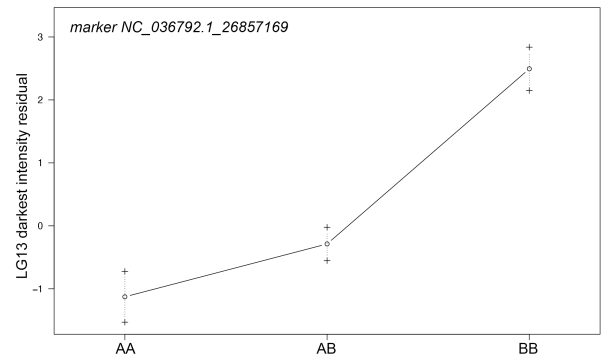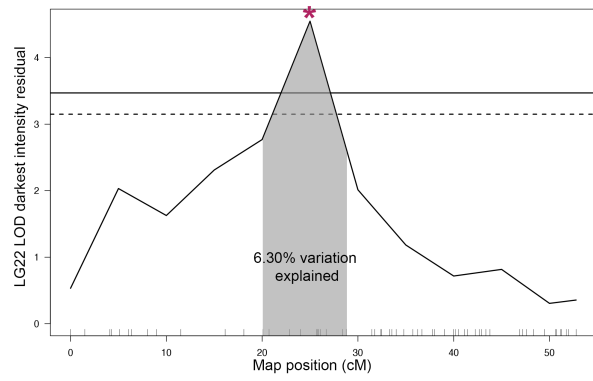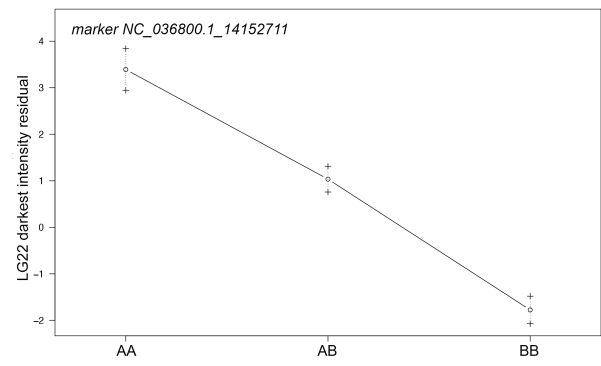

b.

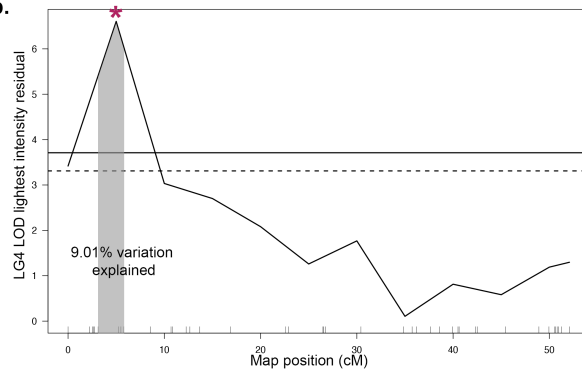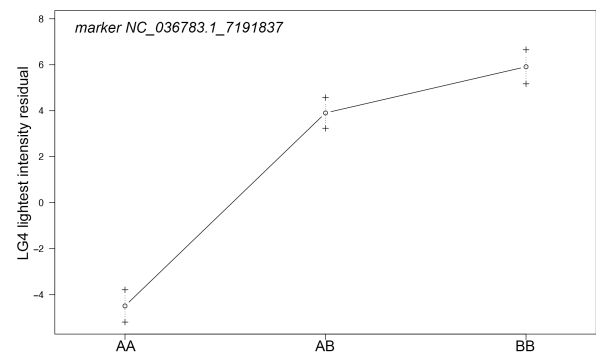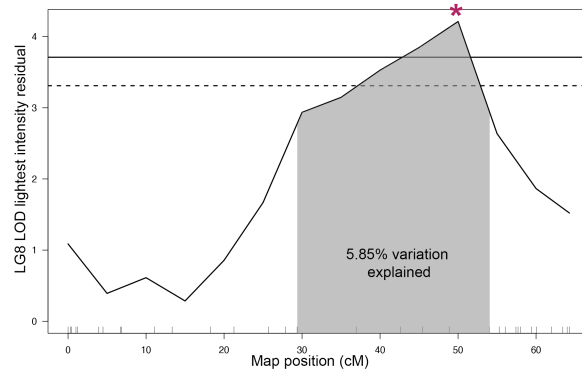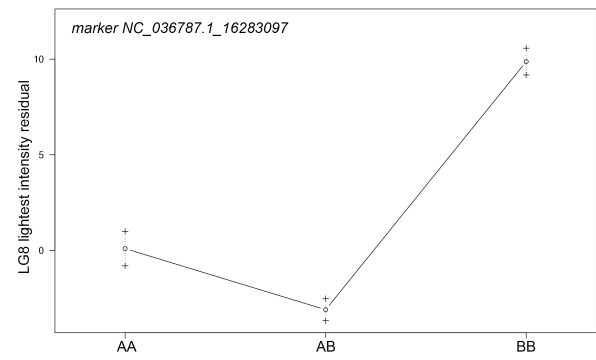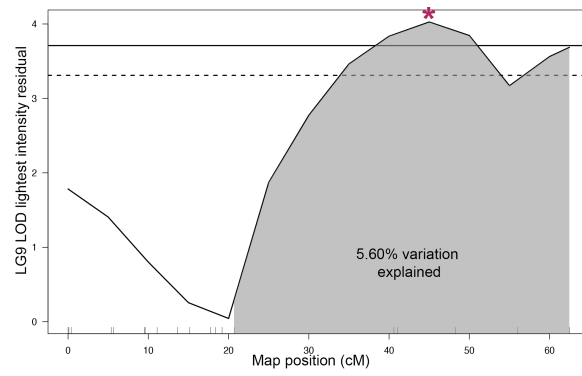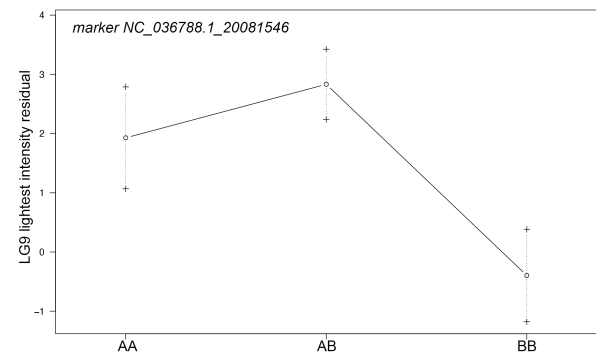

**b.**

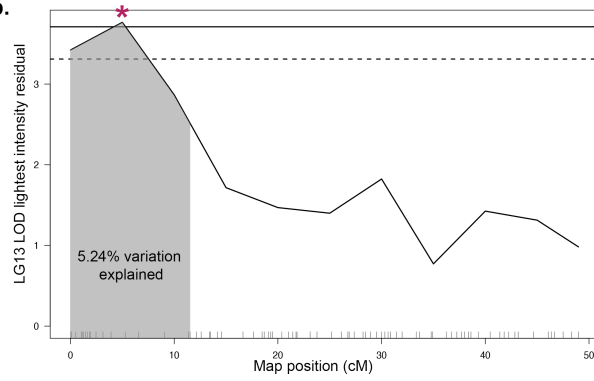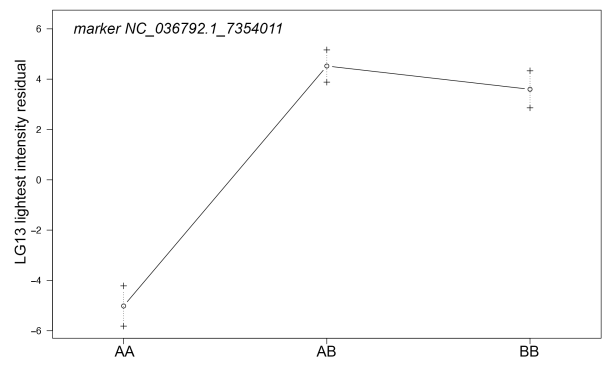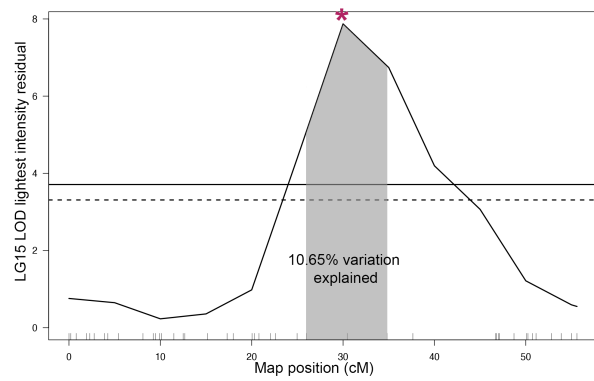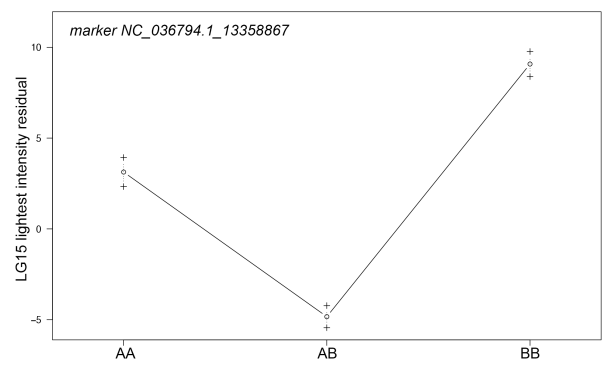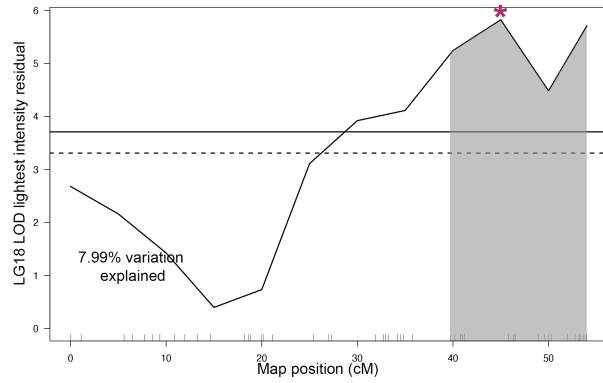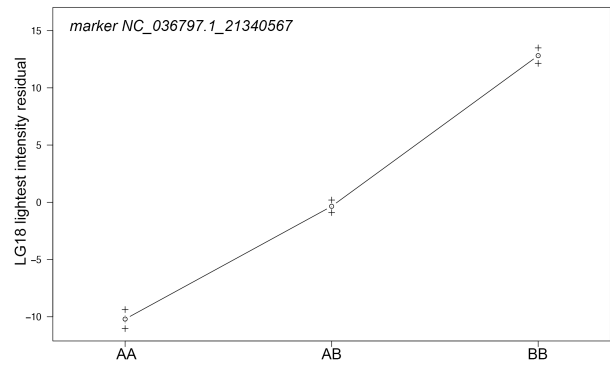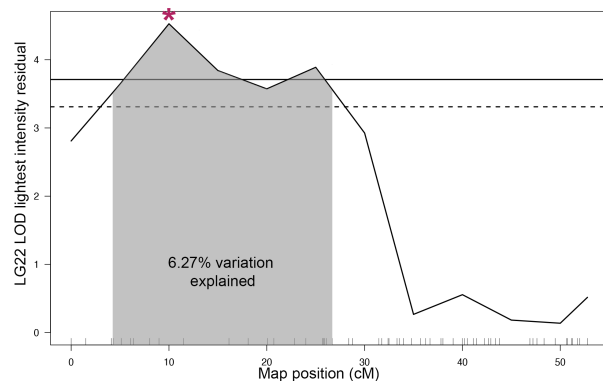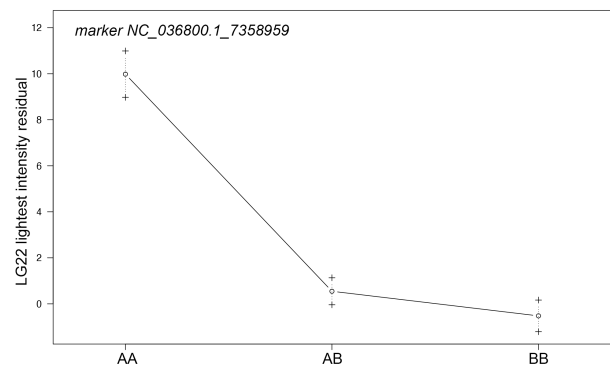

c.

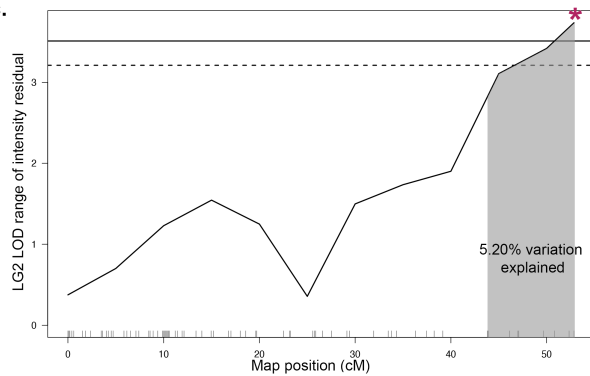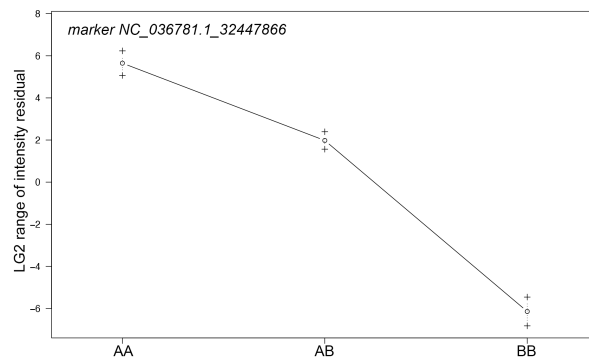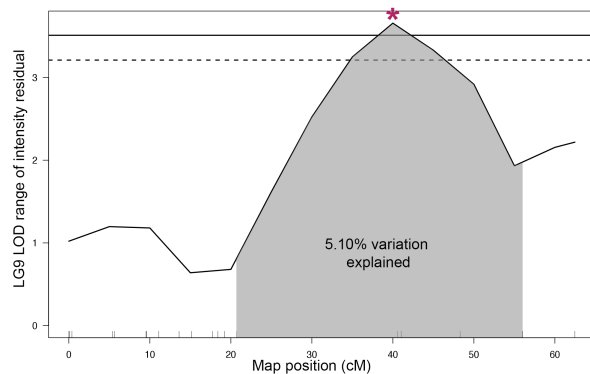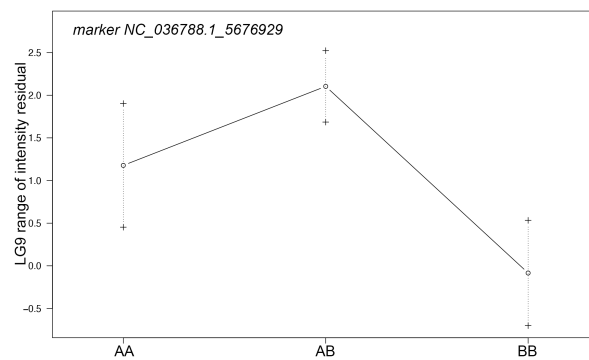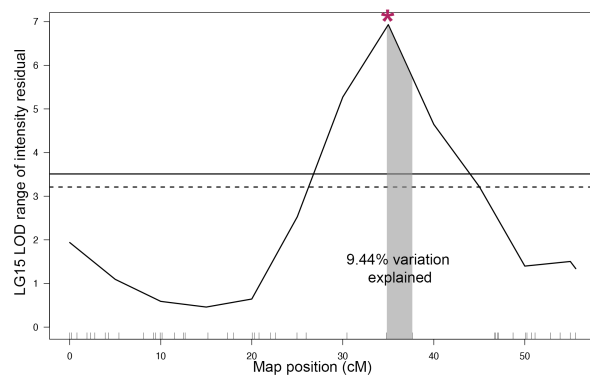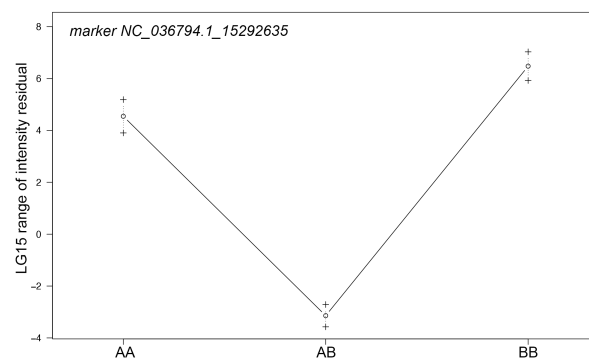

**c.**

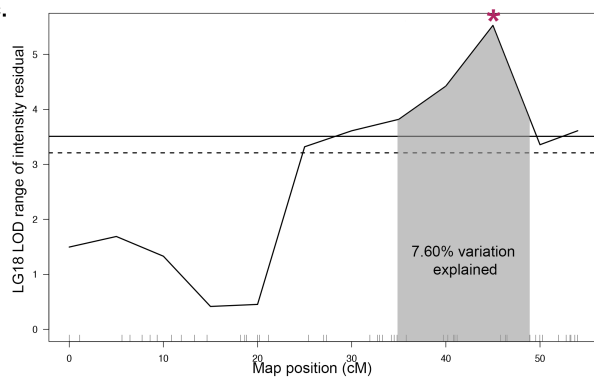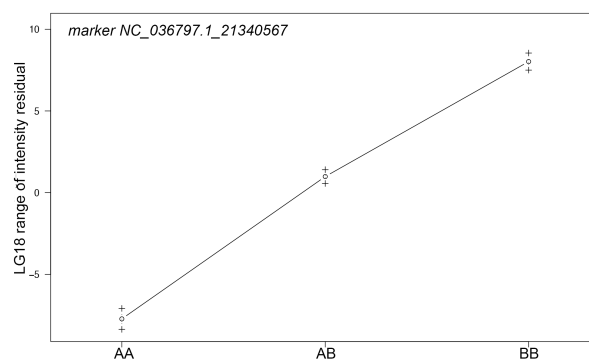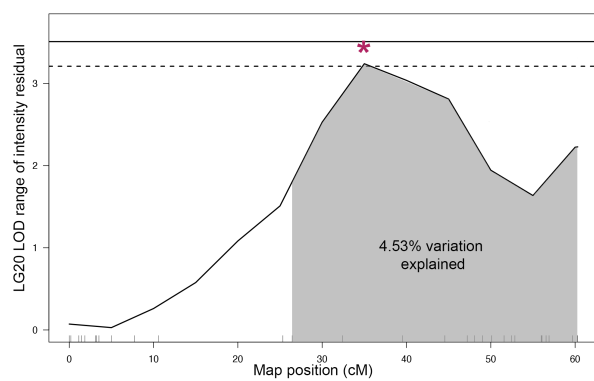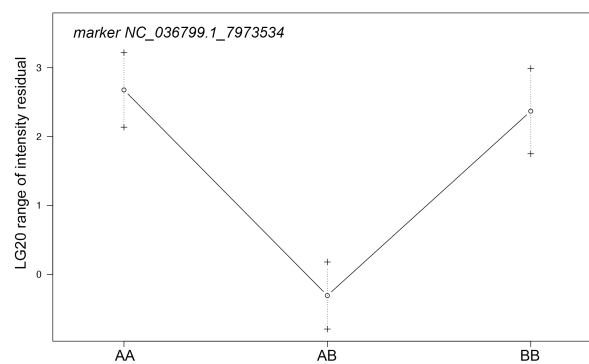

d.

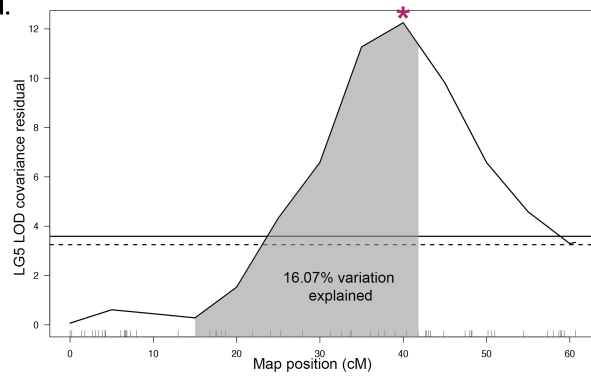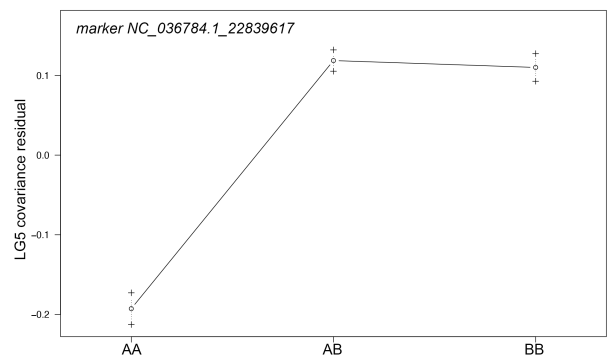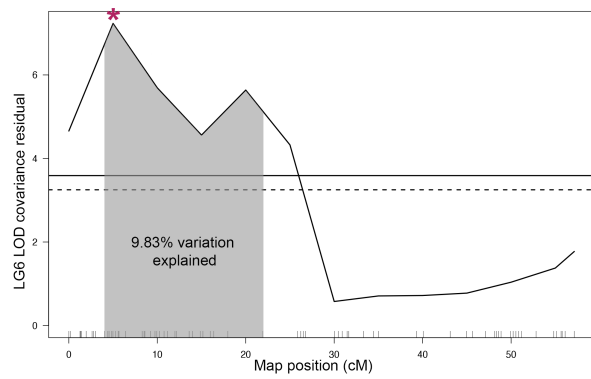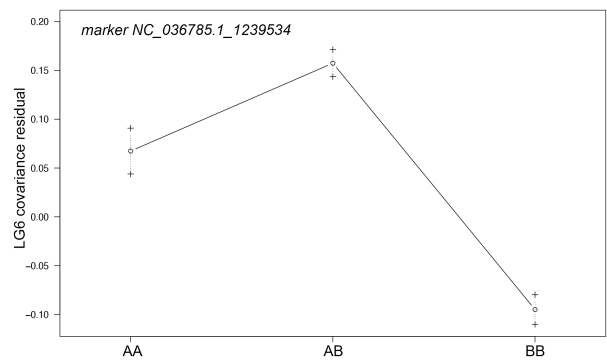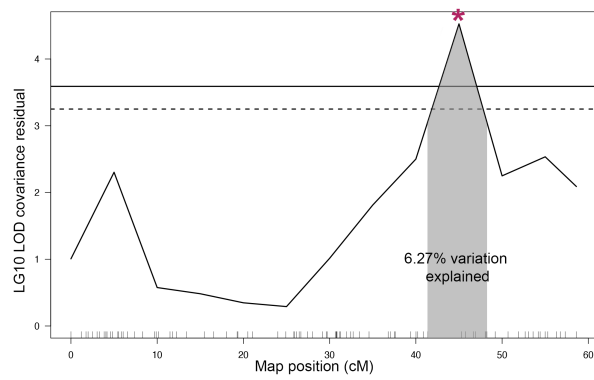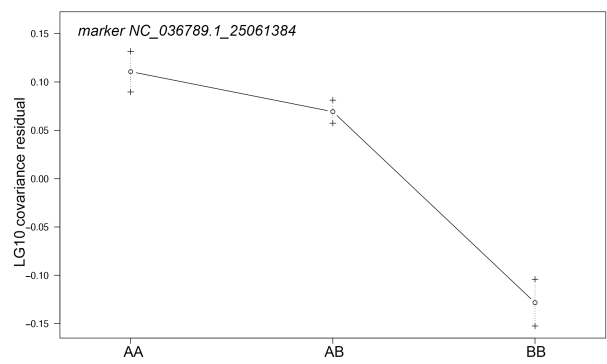

d.

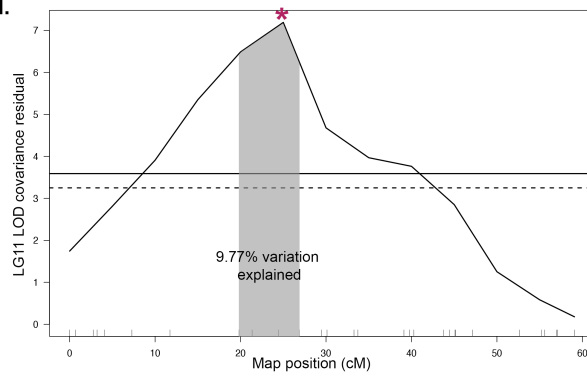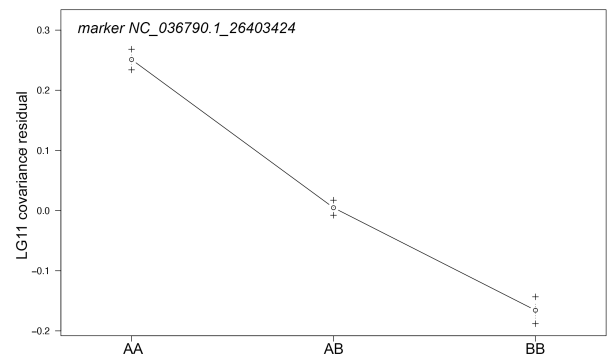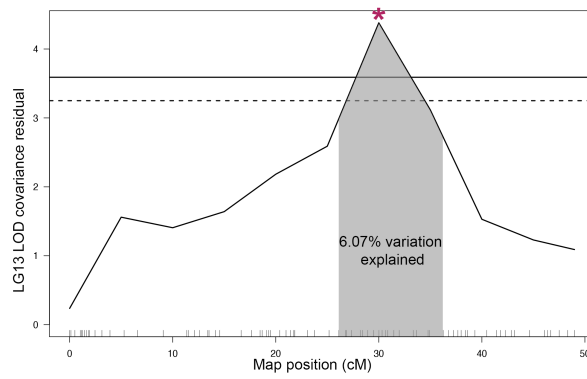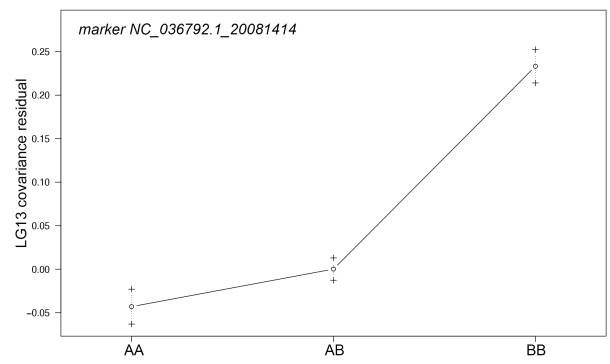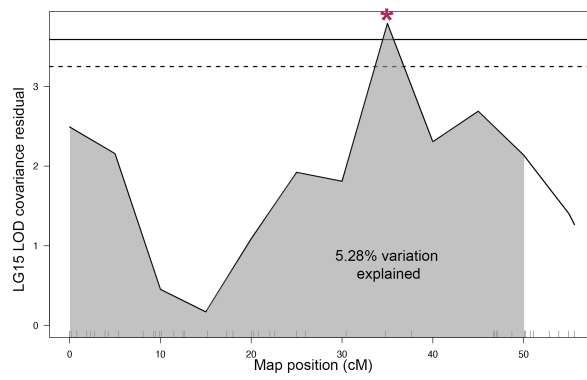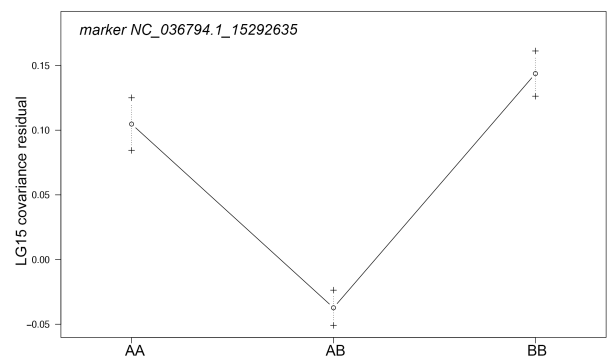

d.

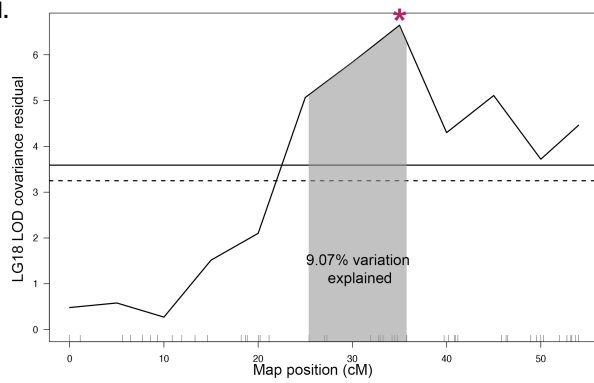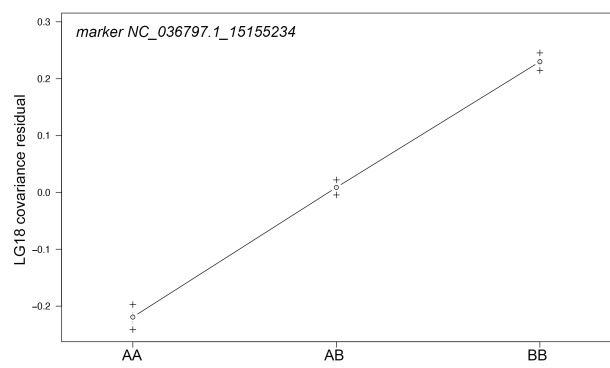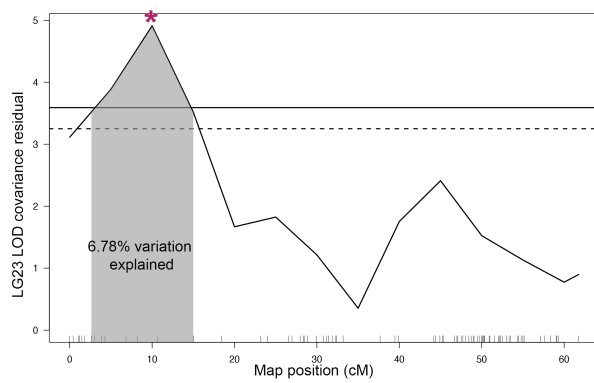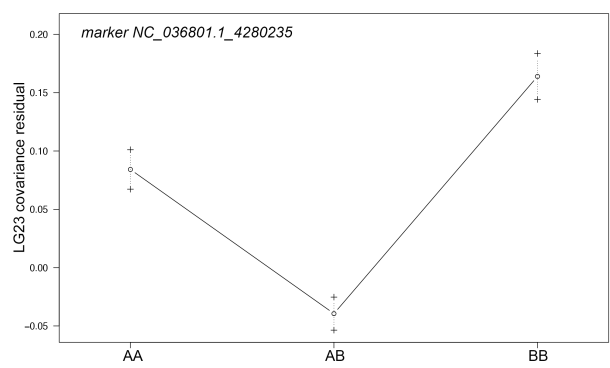

e.

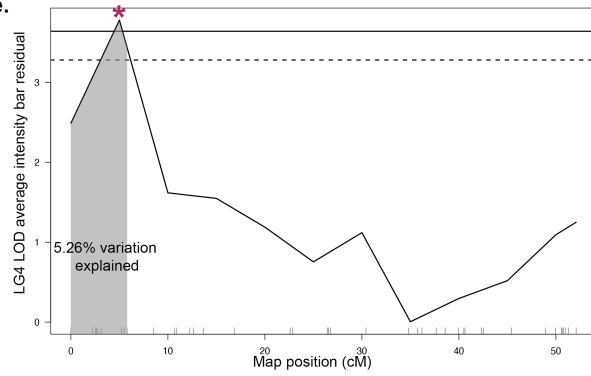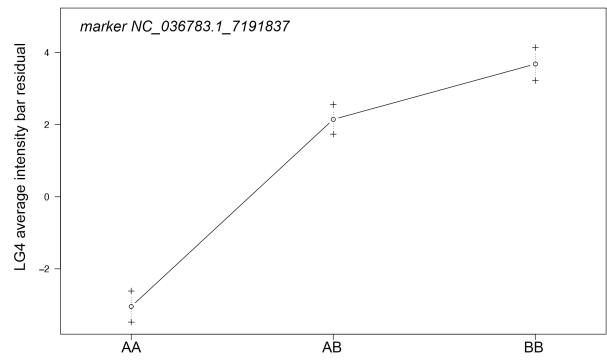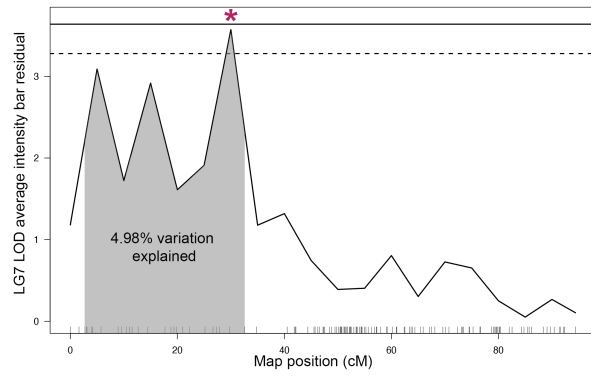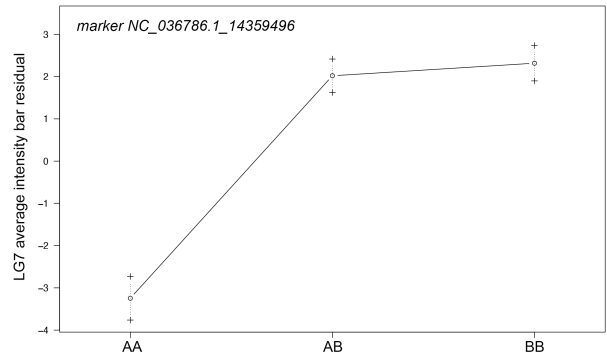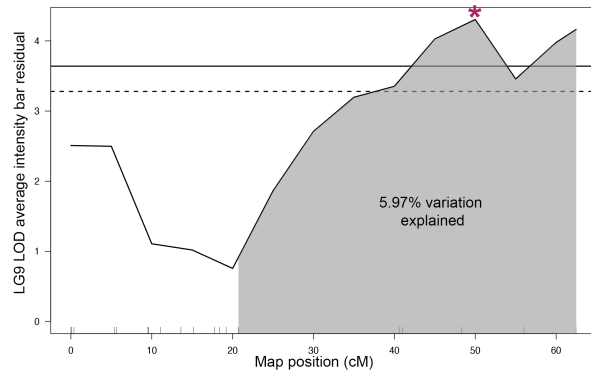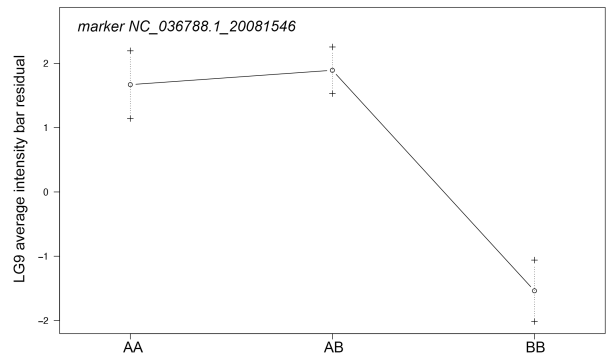

e.

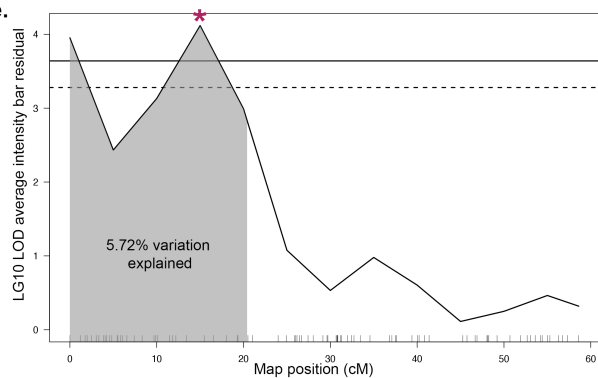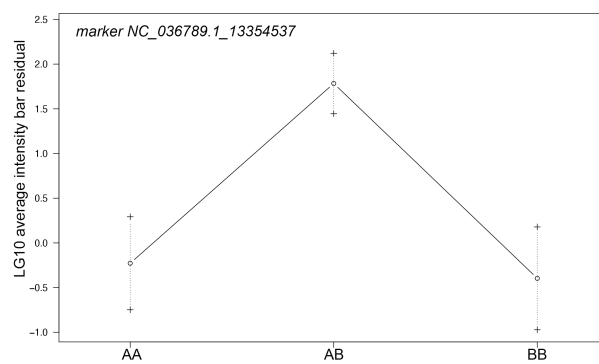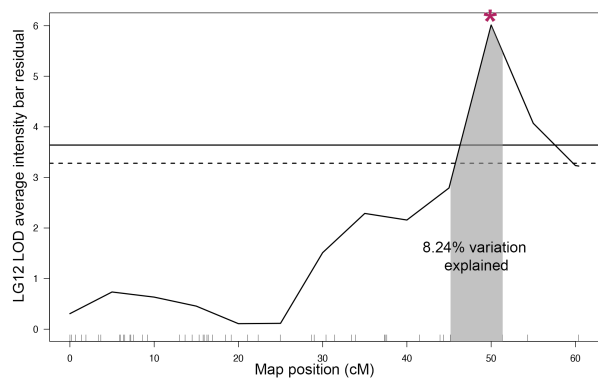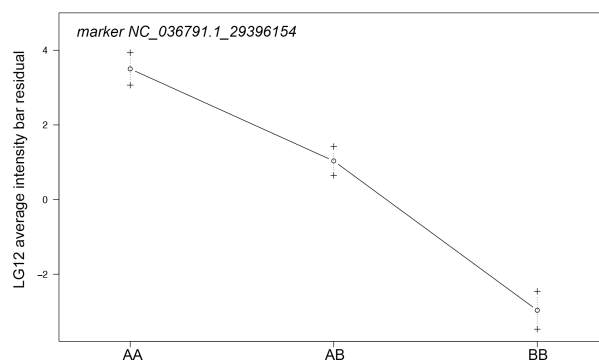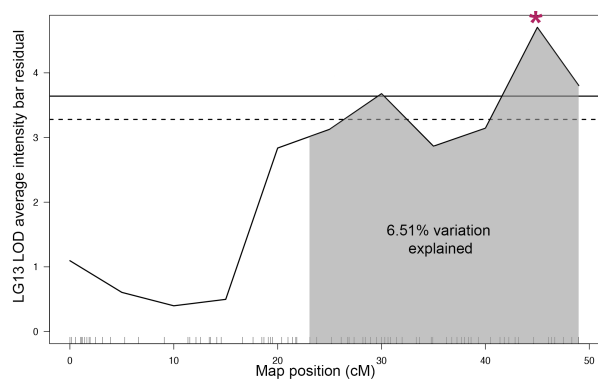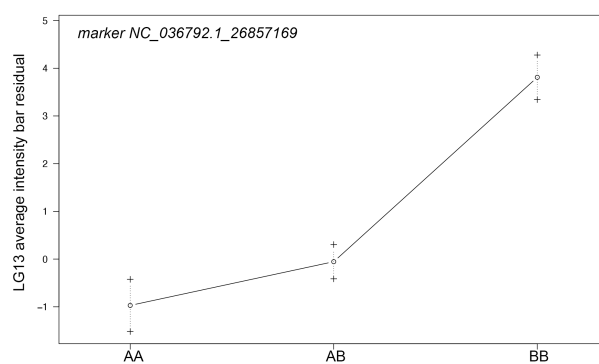

**e.**

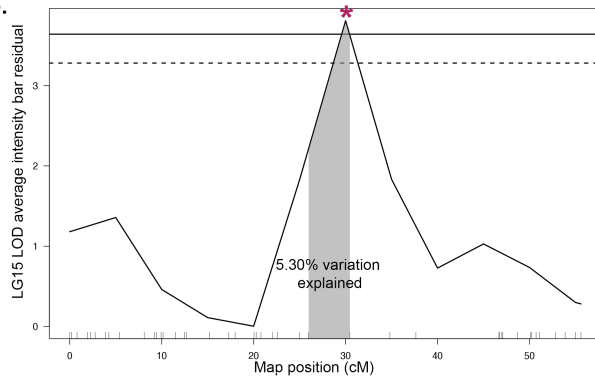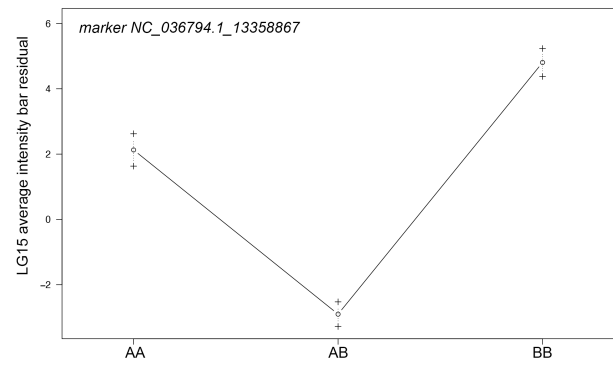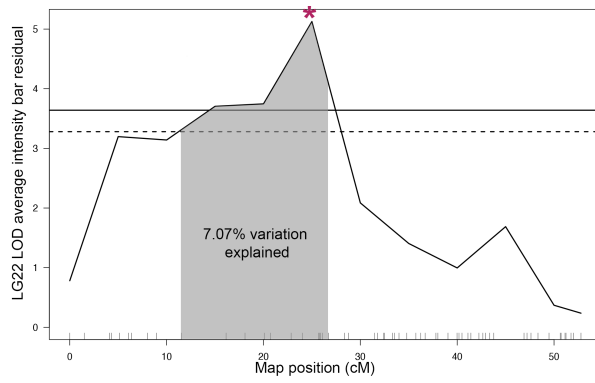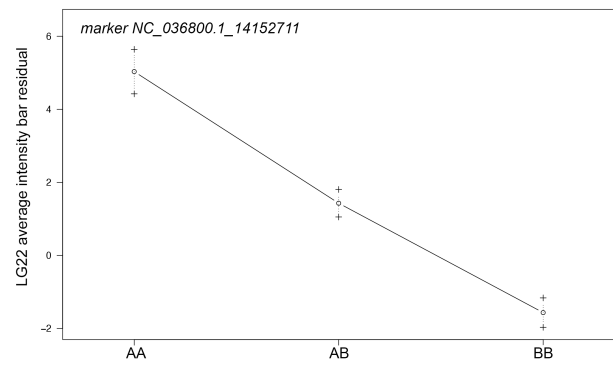

**f.**

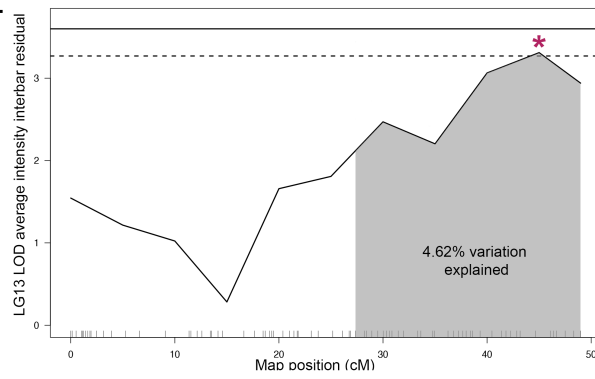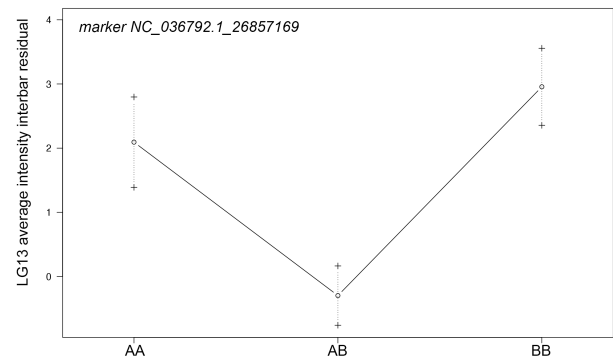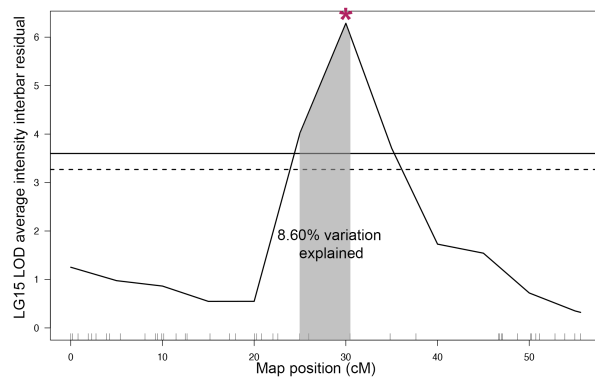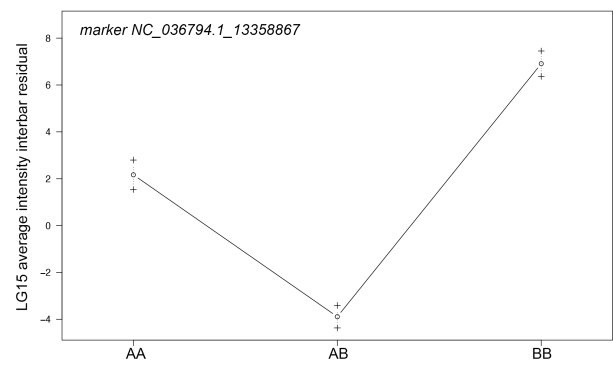

f.

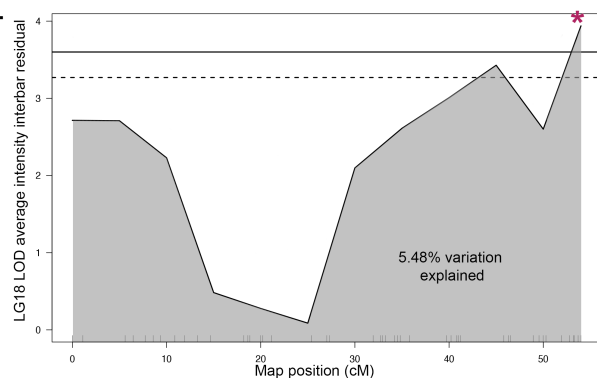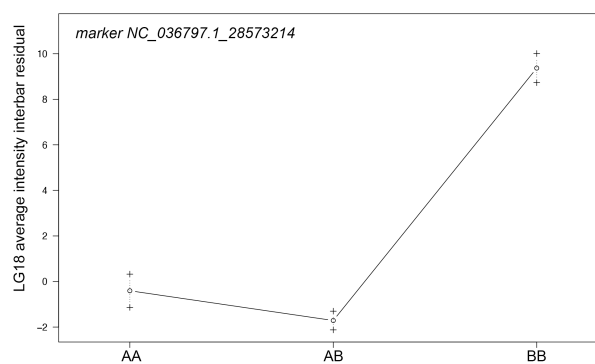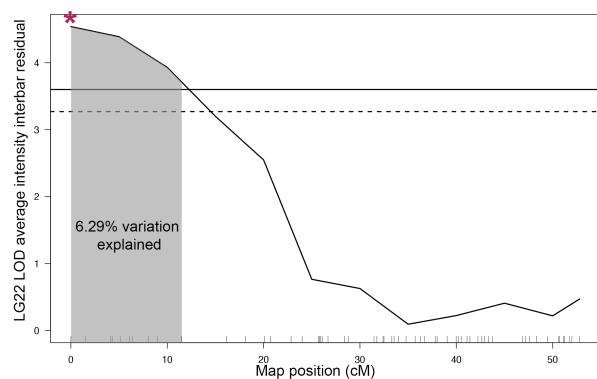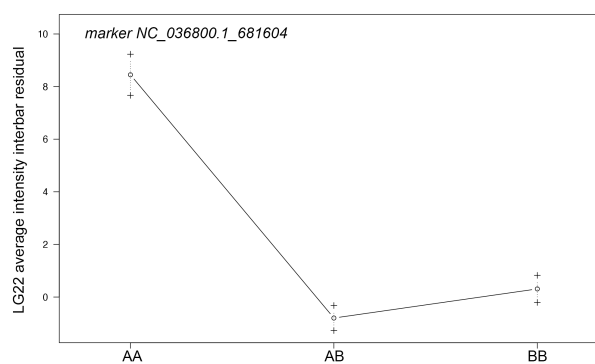

g.

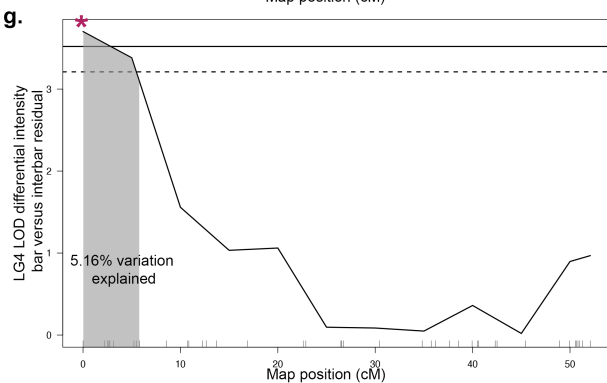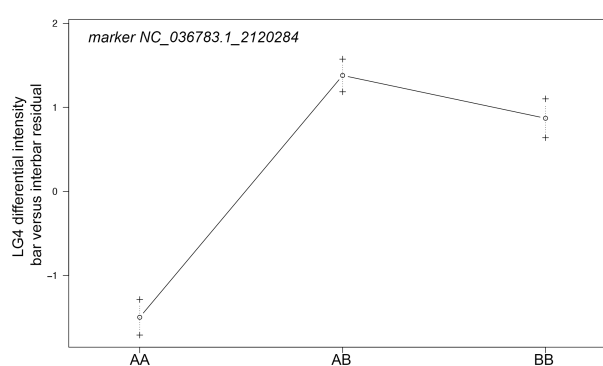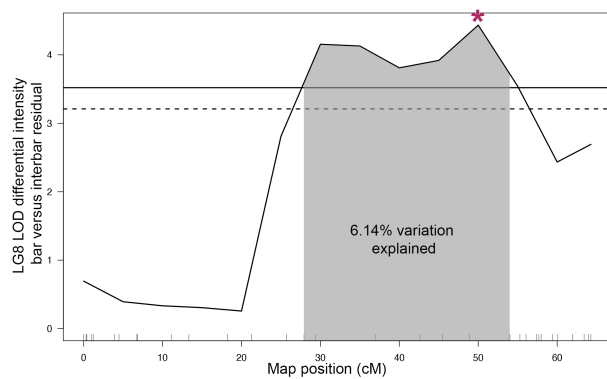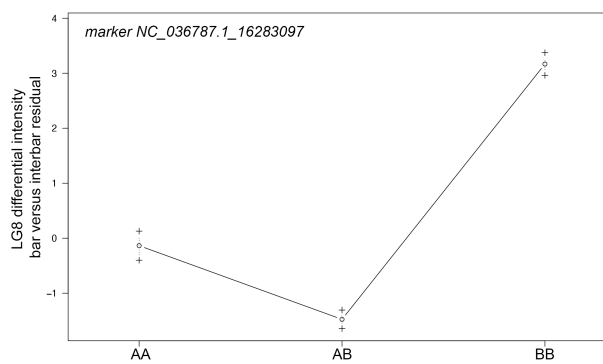

g.

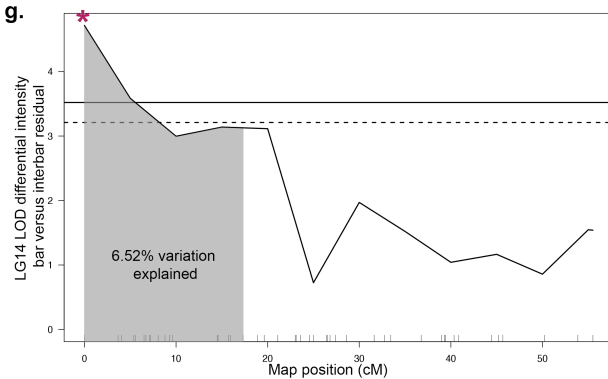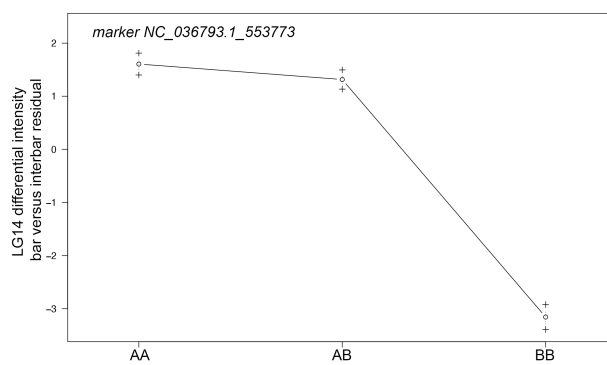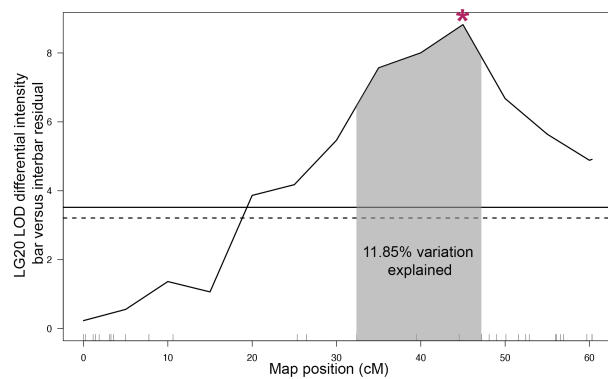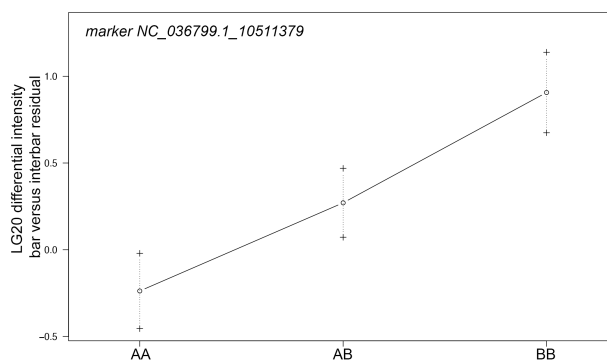

h.

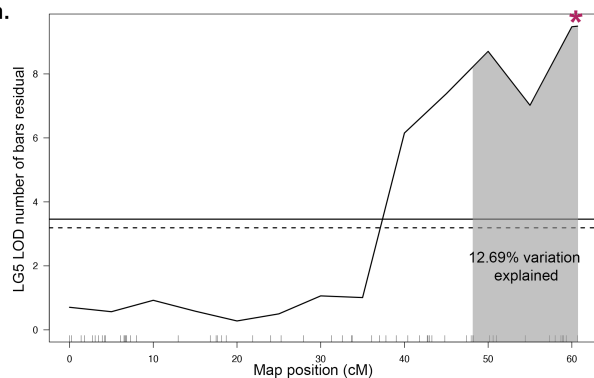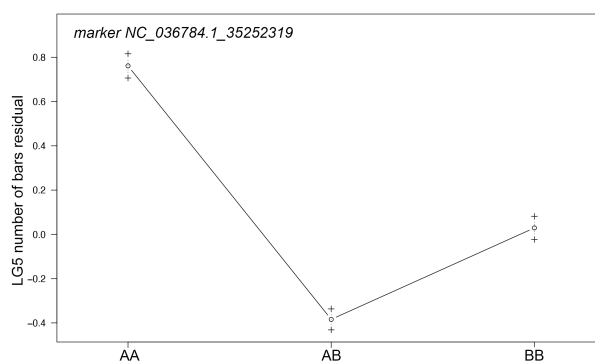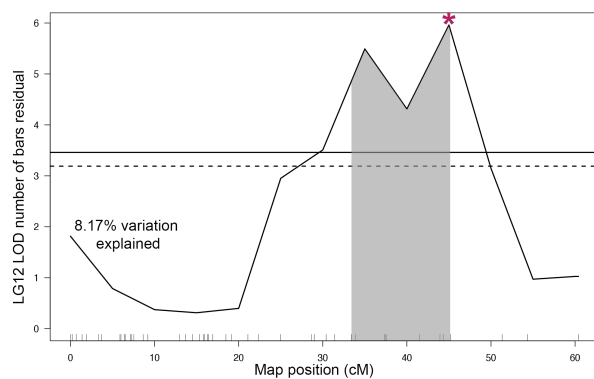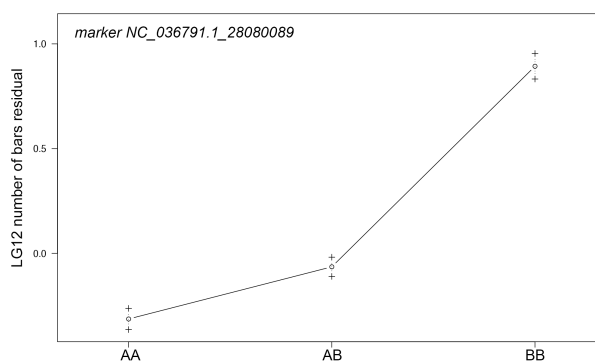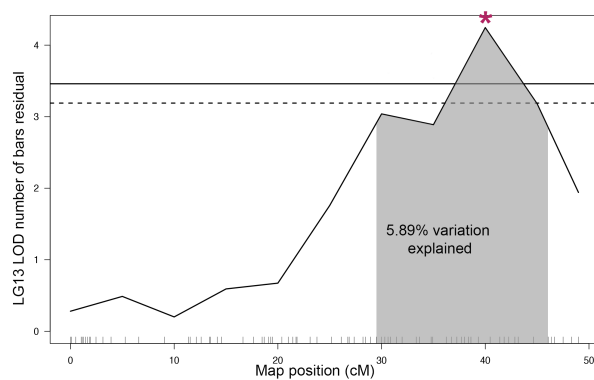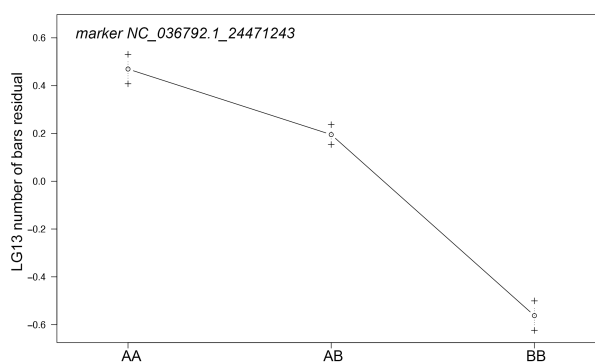

h.

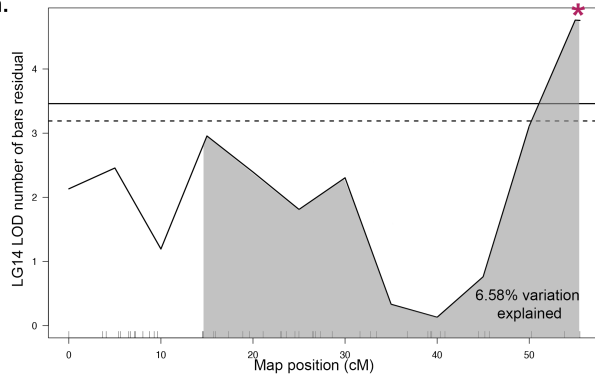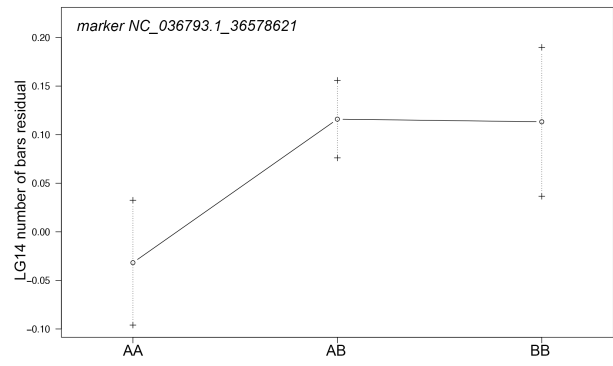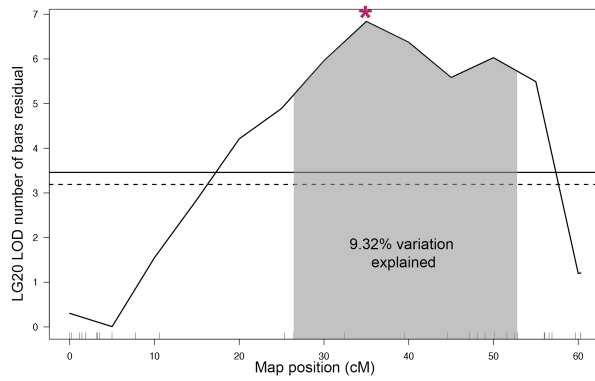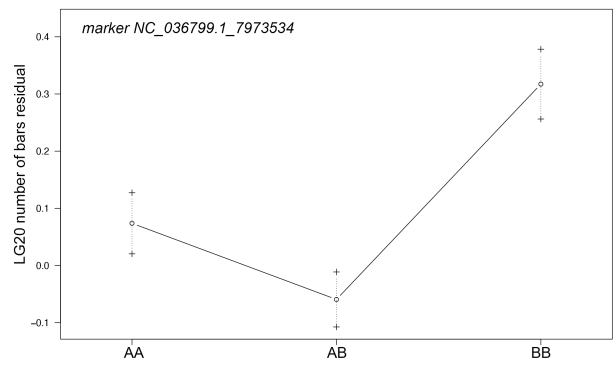

i.

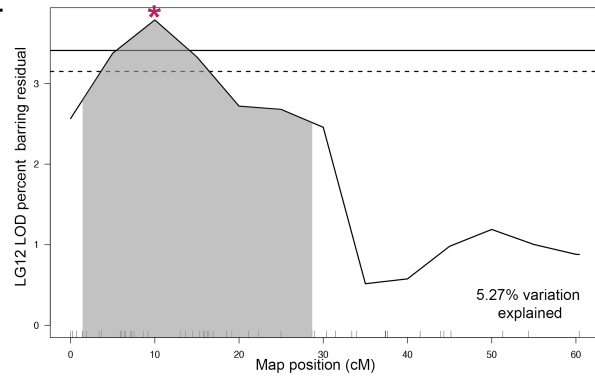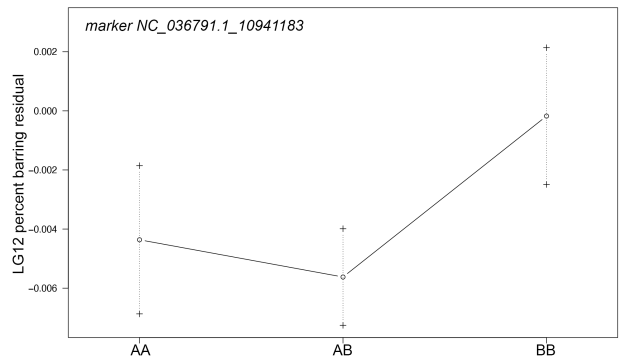

j.

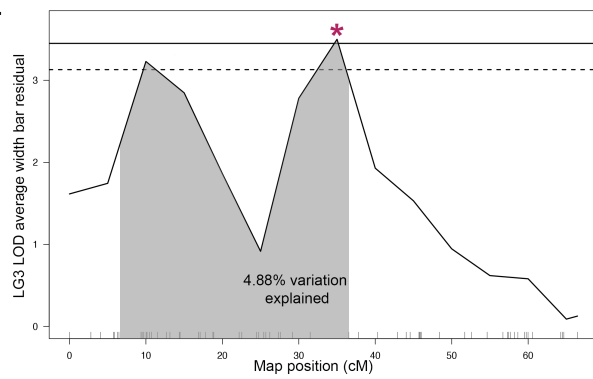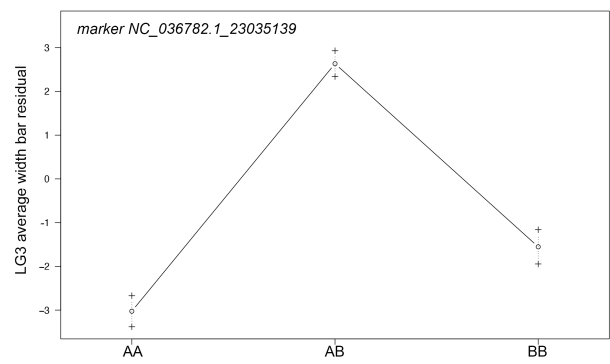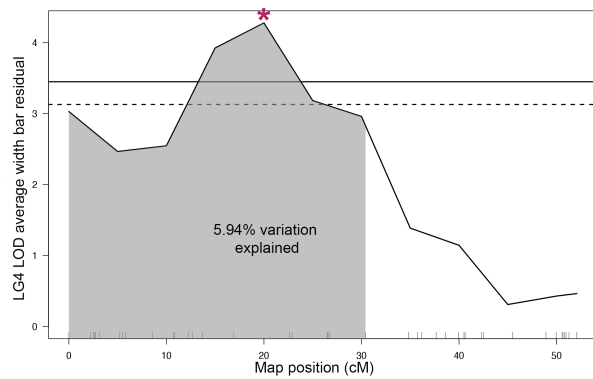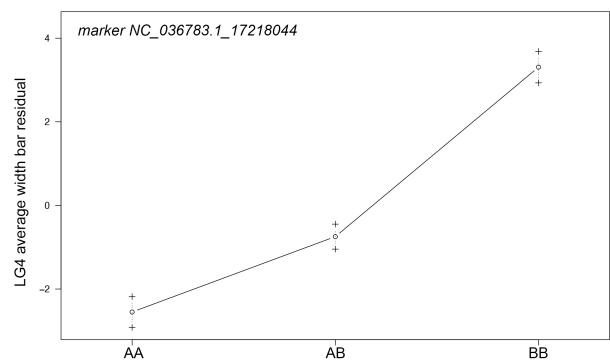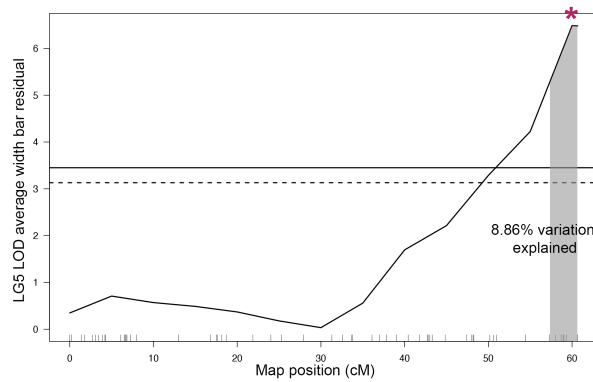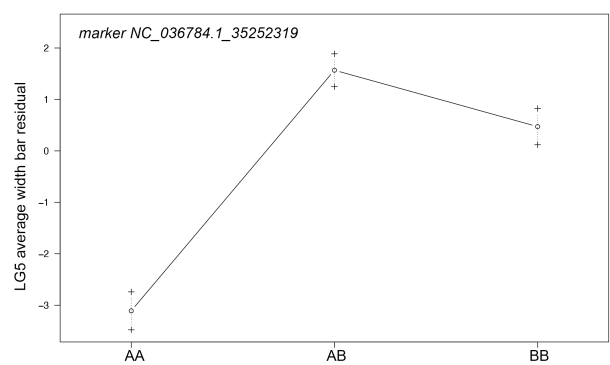

j.

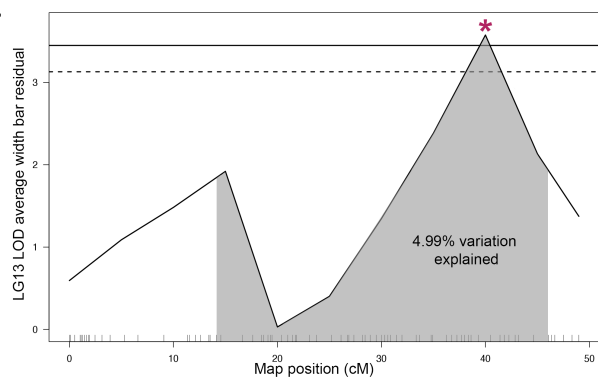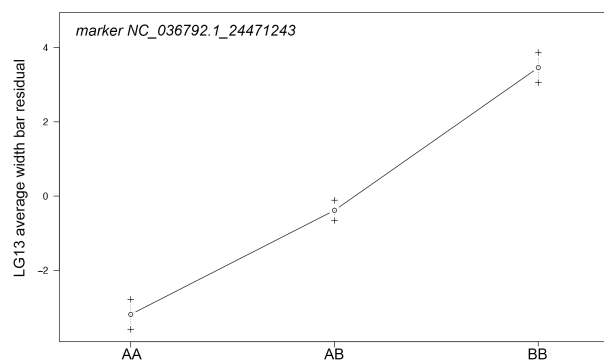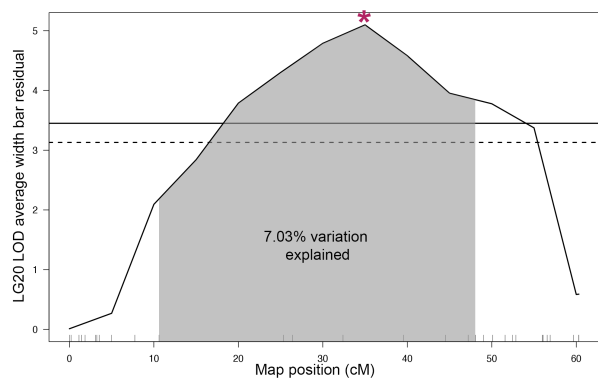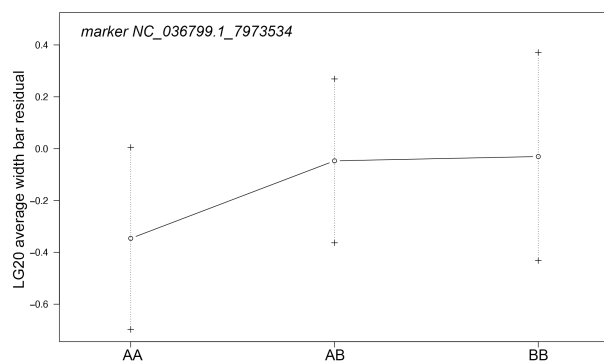

k.

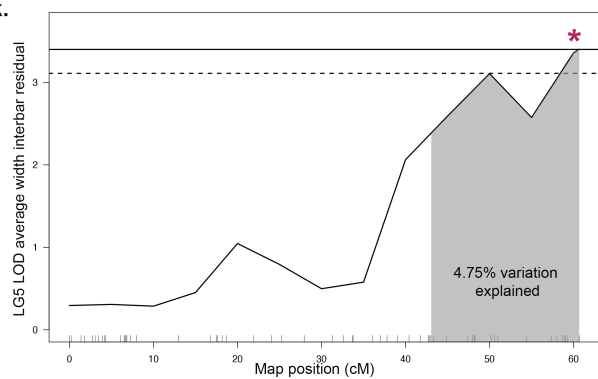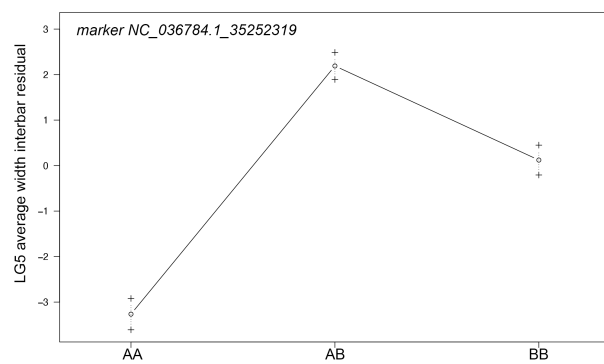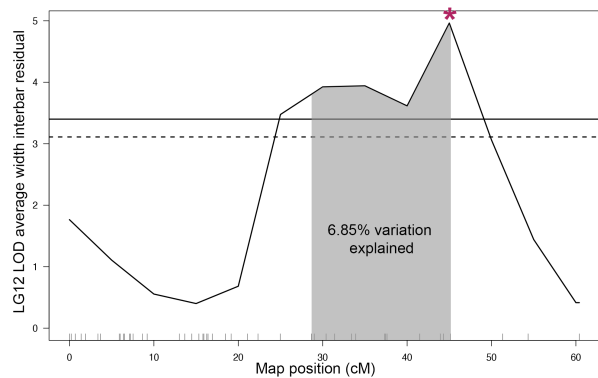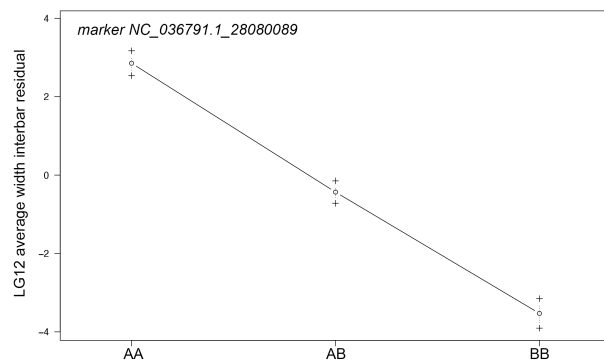

Supplement: S4 Fig — Pigment traits analyzed are residual data for (a) darkest intensity, (b) lightest intensity, (c) range of intensity, (d) covariance, (e) average intensity of bars, (f) average intensity of interbars, (g) differential intensity bars versus interbars, (h) number of bars, (i) percent barring, calculated as sum of total width of bars divided by total width of the isolated region, (j) average width of bars, and (k) average width of interbars. 95% confidence interval for QTL is indicated by shading, percent of total phenotypic variation explained by QTL is reported, and genome-wide significance is shown at the 5% (solid line) and 10% (dashed line) level. Details of QTL scan are in S2 Table and genome-wide visuals are in S3 Fig. Allelic effects are shown for marker at the peak log odds (LOD) score for the QTL, which is indicated by *. The A allele was inherited from the Metriaclima granddam and the B allele from the Aulonocara grandsire. (PDF) [file pone.0306614.s004.pdf]
