# Supplementary material for: Distinct genetic origins of eumelanin levels and barring patterns in cichlid fishes
Source: PLoS One. 2024 Jul 8;19(7):e0306614. doi: 10.1371/journal.pone.0306614 (PMC11230561; doi:10.1371/journal.pone.0306614)
Supplement: S1 Table — Mass was measured immediately upon sacrifice. As described in methods, the standard length (distance from tip of snout to the caudal peduncle) was measured as number of pixels from images and then converted to cm using a ruler in the image. Mean values and standard deviations are reported. Statistical comparisons are based on ANOVA analysis, followed by Tukeys HSD test. (PDF) [file pone.0306614.s005.pdf]

	***Aulonocara* parental**	***Metriaclima* parental**	**F_2 hybrids**	**Statistical significance**
**Body mass (g)**	1.14 ± 0.42	1.99 ± 0.39	1.49 ± 1.01	*Aulonocara* vs *Metriaclima*: p= 0.0344<br>*Aulonocara* vs F_2: p= 0.132<br>*Metriaclima* vs F_2: p= 0.235
**Standard length (cm)**	3.81 ± 0.41	4.48 ± 0.27	4.01 ± 0.91	*Aulonocara* vs *Metriaclima*: p= 0.0687<br>*Aulonocara* vs F_2: p= 0.250<br>*Metriaclima* vs F_2: p= 0.271
